# The MCU and MCUb amino-terminal domains tightly interact: mechanisms for low conductance assembly of the mitochondrial calcium uniporter complex

**DOI:** 10.1016/j.isci.2024.109699

**Published:** 2024-04-10

**Authors:** Megan Noble, Danielle M. Colussi, Murray Junop, Peter B. Stathopulos

**Affiliations:** 1 Department of Physiology and Pharmacology, Schulich School of Medicine and Dentistry, University of Western Ontario, London, ON N6A5C1, Canada; 2 Department of Biochemistry, Schulich School of Medicine and Dentistry, University of Western Ontario, London, ON N6A5C1, Canada

**Keywords:** Cell biology, Structural biology

## Abstract

The mitochondrial calcium (Ca^2+^) uniporter (MCU) complex is regulated via integration of the MCU dominant negative beta subunit (MCUb), a low conductance paralog of the main MCU pore forming protein. The MCU amino (N)-terminal domain (NTD) also modulates channel function through cation binding to the MCU regulating acidic patch (MRAP). MCU and MCUb have high sequence similarities, yet the structural and functional roles of MCUb-NTD remain unknown. Here, we report that MCUb-NTD exhibits α-helix/β-sheet structure with a high thermal stability, dependent on protein concentration. Remarkably, MCU- and MCUb-NTDs heteromerically interact with ∼nM affinity, increasing secondary structure and stability and structurally perturbing MRAP. Further, we demonstrate MCU and MCUb co-localization is suppressed upon NTD deletion concomitant with increased mitochondrial Ca^2+^ uptake. Collectively, our data show that MCU:MCUb NTD tight interactions are promoted by enhanced regular structure and stability, augmenting MCU:MCUb co-localization, lowering mitochondrial Ca^2+^ uptake and implicating an MRAP-sensing mechanism.

## Highlights

• MCUb-NTD exhibits protein concentration-dependent structure and stability
• MCUb-NTD and MCU-NTD heterodimerize with ∼nM affinity in an MRAP-dependent manner
• MCUb-NTD deletion disrupts co-localization with MCU in mitochondria
• MCUb-NTD is necessary for effective inhibition of mitochondrial Ca^2+^ uptake

## Graphical abstract

**Figure:**
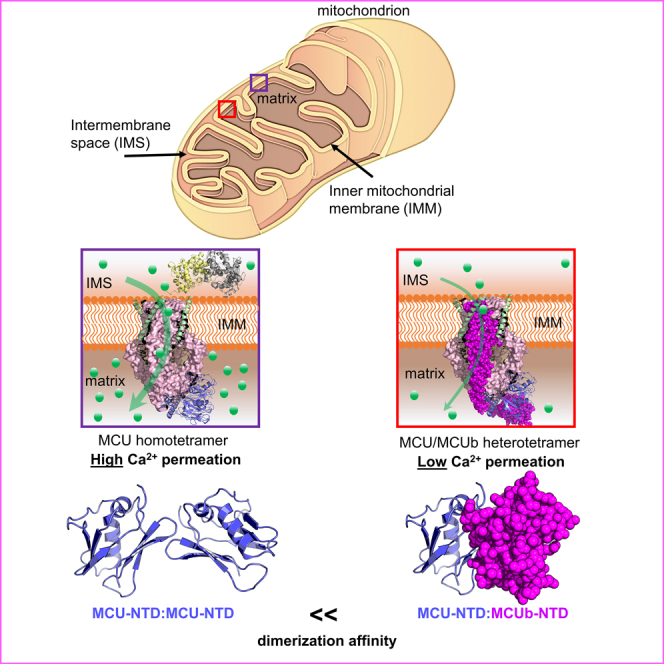

## Introduction

Mitochondria take up calcium (Ca^2+^) to regulate cellular bioenergetics and cell death pathways.^1^^,^^2^^,^^3^ While Ca^2+^ readily diffuses across the outer mitochondrial membrane (OMM) through voltage dependent anion channels (VDAC),^4^ Ca^2+^ uptake across the inner mitochondrial membrane (IMM) into the matrix is precisely controlled by the mitochondrial Ca^2+^ uniporter complex (mtCU).^5^ Under resting cytosolic Ca^2+^ concentrations, mtCU does not transport Ca^2+^.^6^^,^^7^ However, following a rise in cytosolic Ca^2+^ levels, mtCU provides a rapid path for Ca^2+^ movement into the matrix, driven by the large IMM potential (ΔΨ_m_ ∼ -180 mV).^6^^,^^7^

The mtCU forms a Ca^2+^ selective ion channel, comprised of the principal pore-forming mitochondrial Ca^2+^ uniporter (MCU) subunits^5^^,^^8^^,^^9^^,^^10^^,^^11^ and several protein binding partners involved in regulating channel activity [reviewed in^12^^,^^13^^,^^14^. The most prominent regulators include mitochondrial Ca^2+^ uptake-1, -2 and -3 (MICU1, MICU2 and MICU3),^15^^,^^16^^,^^17^^,^^18^ the essential MCU regulator (EMRE),^19^ MCU regulator-1 (MCUR1)^20^ and the MCU dominant-negative beta subunit (MCUb).^21^ MICU1 was the first component of mtCU discovered and functions synergistically with MICU2^17^ as gatekeepers of mtCU.^22^^,^^23^^,^^24^^,^^25^ MICU3 functions in place of MICU2 in skeletal muscle and the central nervous system.^16^ EMRE, a metazoan specific protein, spans the IMM and interacts with both MCU and the MICU1/MICU2 heterodimer,^11^^,^^19^ bridging the Ca^2+^ sensing role of MICU1/MICU2 to the channel activity of MCU.^6^^,^^11^ MCUR1 is a positive regulator of MCU that functions to stabilize the MCU and EMRE interaction.^26^ Lastly, MCUb, an MCU paralog, functions as a negative regulator of mtCU, decreasing mitochondrial Ca^2+^ uptake.^21^^,^^27^^,^^28^^,^^29^

**Figure 1:**
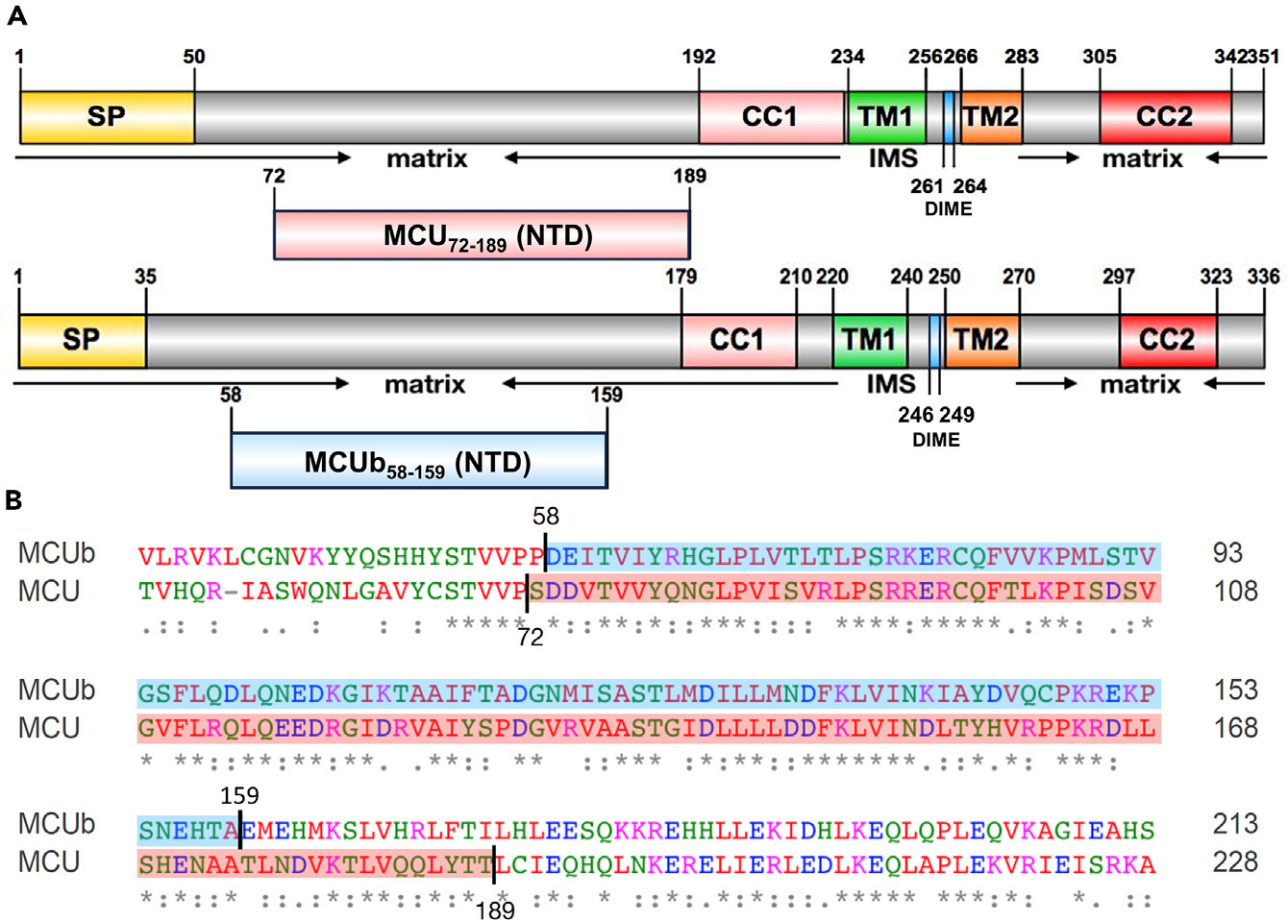
Domain architecture and MCU- and MCUb-NTD sequence alignment (A) MCU and MCUb domain architecture. The location of the signal peptide (SP, yellow), two transmembrane domains (TM1, green; TM2, orange), two coiled-coil domains (CC1, pink; CC2, red), and DIME motif (blue) are shown relative to the N- and C-termini for each construct. The MCU-NTD (pink) and MCUb-NTD (blue) constructs are shown relative to overall domain architecture. (B) Primary structure alignment of MCU- and MCUb-NTDs. Homo sapiens MCU (H.MCU, GenBank: NP_612366.1) and Homo sapiens MCUb (H.MCUb, GenBank: NP_060388.2) were aligned in Clustal Omega.^31^ Fully conserved (∗), highly conserved (:), and partially conserved (.) positions are shown below the respective residue. Residues included in the MCU-NTD and MCUb-NTD constructs are shaded pink and blue, respectively.

Like MCU, MCUb contains two transmembrane domains, two coiled-coil domains and matrix-oriented amino (N)- and carboxyl (C)- terminal domains (NTD/CTD)^21^^,^^30^ (Figure 1A). Despite sharing ∼49% sequence identity with MCU, MCUb is unable to form a Ca^2+^ permeable pore when reconstituted in lipid bilayers.^21^ Further, MCUb directly interacts with MCU, resulting in lower mtCU Ca^2+^ conductance.^19^^,^^21^^,^^27^ The inhibitory effect may be due to changes in Ca^2+^ permeation properties of the channel, previously attributed to two key amino acid substitutions in MCUb compared to MCU (i.e., R252W and E257V – human MCU residue numbering)^21^ or altered pore conformation.^28^^,^^29^^,^^32^ Additionally, MCUb does not interact with MICU1,^27^^,^^29^ which may negatively impact channel gating and be a consequence of altered MCUb-EMRE interactions.^28^

While there are several proteins that regulate MCU channel activity, the MCU-NTD also functions as an autoregulatory domain within MCU channel subunits. Specifically, the MCU-NTD contains divalent cation binding and post-translational modification sites, which influence channel function.^33^^,^^34^^,^^35^^,^^36^^,^^37^^,^^38^^,^^39^ Interestingly, *S*-glutathionylation within the MCU-NTD has been shown to promote channel assembly and activation whereas Ca^2+^ and Mg^2+^ binding to the MCU-NTD promotes disassembly and channel inactivation.^28^^,^^33^^,^^34^^,^^35^^,^^36^ Given the ability of MCU-NTD to modulate self-association equilibria and the similarities between MCU and MCUb, we postulated that MCUb-NTD also plays a critical role in mtCU assembly. Thus, we isolated the MCUb-NTD to study the biophysical properties and role in mediating MCU:MCUb interactions.

Here, we reveal that the MCUb-NTD exhibits α-helical and β-sheet like structure, with remarkably high thermal stability. The structure and stability of the MCUb-NTD is highly dependent on protein concentration such that higher concentrations suppress regular secondary structure and decrease thermal stability. Excitingly, we find that the MCU-NTD and MCUb-NTDs heteromerically interact with much higher affinity than homomeric interactions, and the heteromeric interactions are favored by increased secondary structure and stability of both NTDs. Further, we show that upon binding to the MCUb-NTD, the MCU-NTD undergoes structural perturbations in the region critical for divalent cation binding. Finally, we find that deletion of the MCUb-NTD from full-length MCUb, suppresses co-localization with MCU in mammalian cells. Collectively, our data suggests that MCUb has a strong tendency to heteromerically interact with MCU via the NTD, which we propose is critical for integration of MCUb into mtCU and interactions of MCUb with mtCU for lower Ca^2+^ conductance.

## Results

### The MCUb N-terminal domain (NTD) adopts α-helix and β-sheet structure

**Figure 2:**
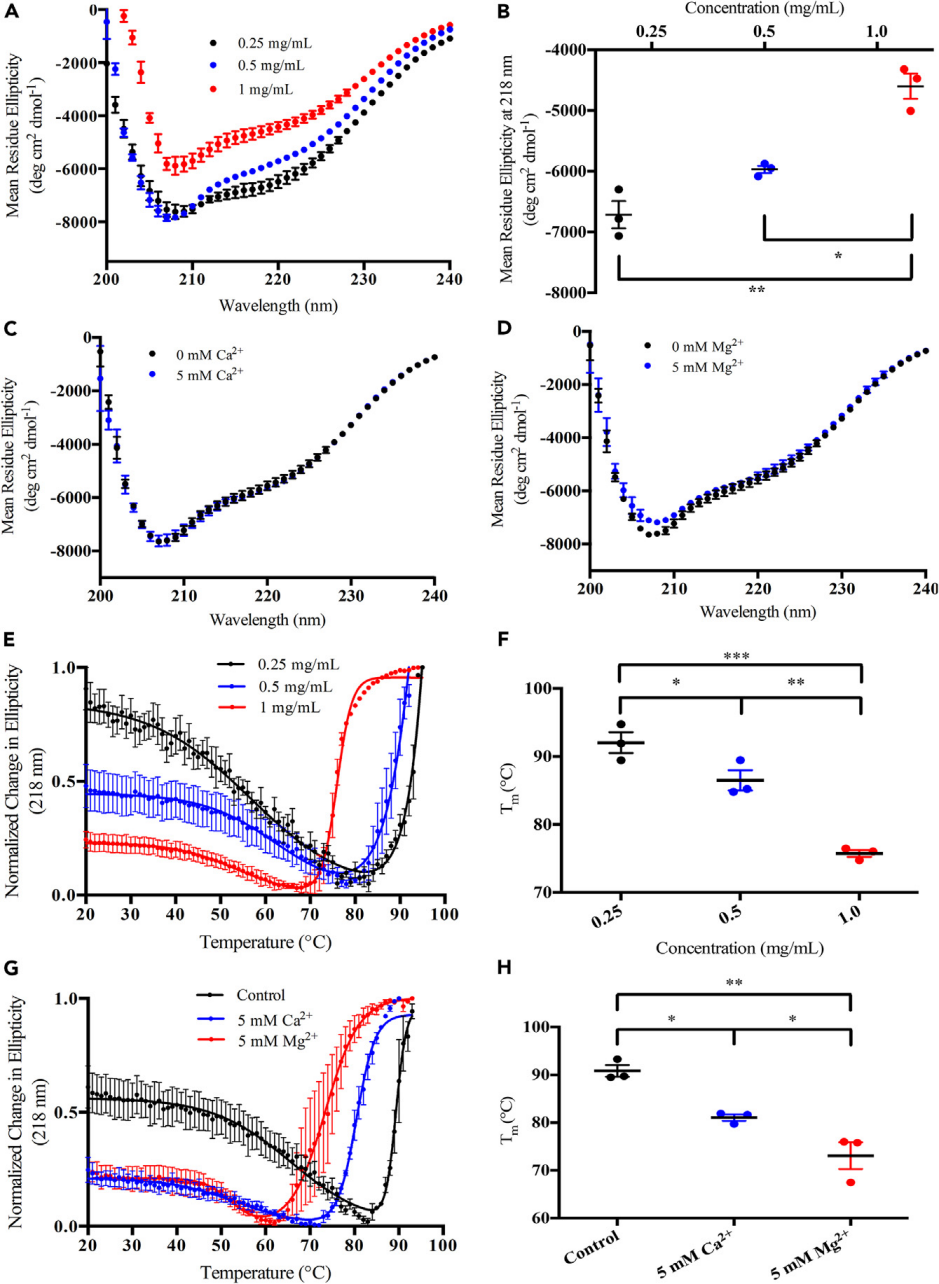
Secondary structure and thermal stability of MCUb-NTD as a function of protein and divalent cation concentration (A) Far-UV CD spectra of MCUb-NTD at 0.25 mg/mL (black circles), 0.5 mg/mL (blue circles) and 1 mg/mL (red circles). (B) Comparison of the mean residue ellipticity (MRE) signal at 218 nm for data shown in A). (C) Far-UV CD spectra of MCUb-NTD at 0.5 mg/mL in the absence (black circles) and presence (blue circles) of 5 mM CaCl_2_. (D) Far-UV CD spectra of MCUb-NTD at 0.5 mg/mL in the absence (black circles) and presence (blue circles) of 5 mM MgCl_2_. (E) Thermal melts of MCUb-NTD at 0.25 mg/mL (black circles), 0.5 mg/mL (blue circles) and 1 mg/mL (red circles). (F) Comparison of the T_m_ values extracted from the data shown in E). (G) Thermal melts of MCUb-NTD at 0.5 mg/mL in the absence (black circles) and presence of 5 mM CaCl_2_ (blue circles) or 5 mM MgCl_2_ (red circles). (H) Comparison of the T_m_ values extracted from the data shown in G). In E) and G), thermal melts were constructed from the change in CD signal at 218 nm as a function of temperature, and solid lines are two transition Boltzmann equation fits to the data. All experiments were carried out in 20 mM Tris (pH 8.5), 150 mM NaCl, 1 mM DTT at 4°C. Data are means ± SEM of *n* = 3 experiments. Statistical analysis was a one-way ANOVA followed by Tukey’s post-hoc test (∗*p* < 0.05, ∗∗*p* < 0.01, ∗∗∗*p* < 0.001). See also Table S1.

Pairwise sequence alignment of the MCU and MCUb NTDs using LALIGN^40^ shows a nearly 80% sequence similarity and 52% sequence identity between the two domains (Figure 1B). Far-UV circular dichroism (CD) spectra at 0.25 mg mL^−1^, 0.5 mg mL^−1^ and 1.0 mg mL^−1^ show one distinct minimum at ∼208 nm and a more subtle minimum, or shoulder, centered at ∼218–222 nm (Figure 2A). These minima indicate a mix of α-helical and β-sheet secondary structure for MCUb-NTD at all three concentrations.^41^ The most negative mean residue ellipticity (MRE) at 208 nm and 218 nm was observed at 0.25 mg mL^−1^, while the least negative MRE was observed at 1.0 mg mL^−1^ (Figure 2A and 2B; Table S1). Thus, lower MCUb-NTD concentrations favor greater levels of α-helical and β-sheet structure. The MRE spectral signal and shape did not significantly change in the presence of 5 mM CaCl_2_ or MgCl_2_ (Figures 2C and 2D; Table S1). Thus, MCUb-NTD primarily folds into α-helical and β-sheet configurations with the level of secondary structure impacted by protein concentration but not divalent cations.

### MCUb-NTD thermal stability is protein concentration and divalent cation dependent

Having found that the lowest protein concentration showed the highest levels of secondary structure, we next assessed the effect of MCUb-NTD concentration on thermal stability. Consistent with the far-UV CD spectra showing the most negative MRE signal, the lowest MCUb-NTD concentration (0.25 mg mL^−1^) exhibited the highest midpoint of temperature denaturation (T_m_) of 92.0 ± 1.5°C (Figures 2E and 2F; Table S1). Remarkably, increasing protein concentration to 0.5 mg mL^−1^ and 1 mg mL^−1^ decreased the stability by ∼5.5°C (T_m_ = 86.5 ± 1.4°C) and ∼16.2°C (T_m_ = 75.8 ± 0.5°C), respectively (Figures 2E and 2F; Table S1). Further, while Ca^2+^ and Mg^2+^ did not affect the far-UV CD spectra of MCUb-NTD at 4°C, both divalent cations significantly altered the thermal stability at 0.5 mg mL^−1^. Specifically, the presence of 5 mM MgCl_2_ or 5 mM CaCl_2_ decreased the stability by ∼16.0°C (T_m_ = 73.4 ± 0.5°C) and ∼8.9°C (T_m_ = 80.5 ± 0.2°C), respectively (Figures 2G and 2H; Table S1). Overall, our data show that the MCUb-NTD is more stable at lower protein concentrations and in the absence of Mg^2+^ and Ca^2+^ divalent cations.

### MCUb-NTD undergoes concentration and divalent cation dependent self-association

To test whether the effects of protein concentration or divalent cations on the secondary structure and stability correlate with MCUb-NTD oligomerization, dynamic light scattering (DLS) was applied to measure the distribution of hydrodynamic radii (R_h_) at 2 mg mL^−1^, 1 mg mL^−1^, and 0.5 mg mL^−1^. Note that during MCUb-NTD purification laddering was observed on sodium dodecyl sulfate polyacrylamide gel electrophoresis (SDS-PAGE) gels, highlighting the ability for higher order oligomerization (Figures S1A and S1B). Congruently, a shift in the distribution of R_h_ to larger sizes was observed as the MCUb-NTD concentration was increased (Figure S1C). At the highest protein concertation (2 mg mL^−1^), a clear bimodal size distribution with baseline separation was noted, whereas both 1 mg mL^−1^ and 0.5 mg mL^−1^ samples displayed overlapping size distributions. Interestingly, the presence of 5 mM CaCl_2_ or 5 mM MgCl_2_ also caused a systematic shift in the distribution of R_h_ to larger sizes (Figures S1D–S11F). MgCl_2_ promoted distinct size distributions with baseline separation at 0.5 mg mL^−1^, 1 mg mL^−1^, and 2 mg mL^−1^ MCUb-NTD, while CaCl_2_ promoted distinct size distributions with baseline separation only at 2 mg mL^−1^ MCUb-NTD. Together, our DLS data show that higher protein concentrations and the presence of ∼mM levels of divalent cations promote larger MCUb-NTD oligomers.

### MCUb-NTD undergoes a concentration and divalent cation dependent increase in solvent exposed hydrophobicity

**Figure 3:**
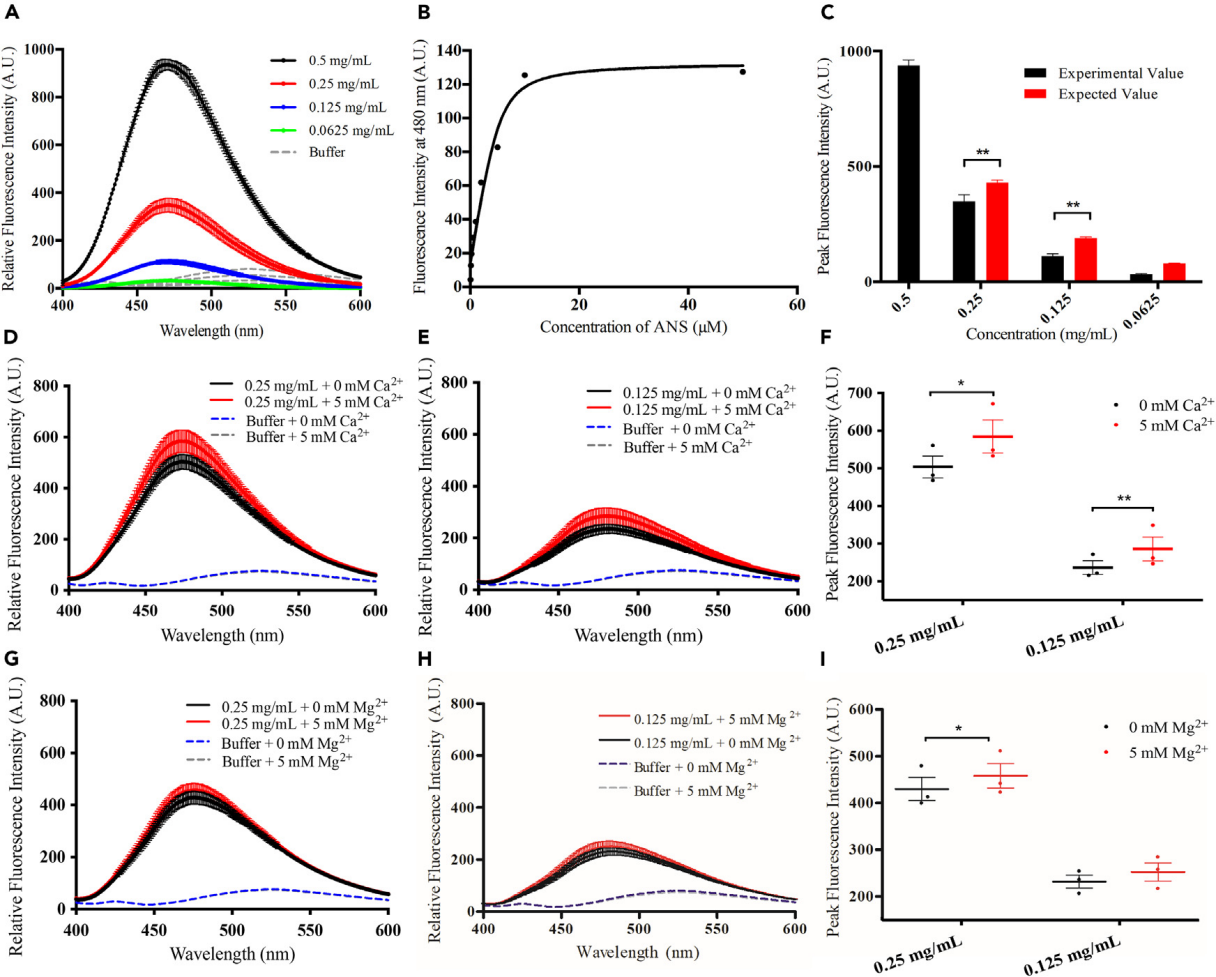
ANS binding to MCUb-NTD as a function of protein and divalent cation concentration (A) ANS fluorescence emission spectra in the presence of 0.5 mg/mL (black), 0.25 mg/mL (red), 0.125 mg/mL (blue), or 0.0625 mg/mL (green) MCUb-NTD. The ANS:protein monomer molar ratio was constant by using doubling dilutions with an ANS-free buffer. The ANS fluorescence emission spectra at 50, 25, 12.5, 6.25 μM in the absence of protein are shown for reference (gray). (B) Representative titration of ANS binding to MCUb-NTD. ANS fluorescence emission spectra acquired in the absence of protein were subtracted from spectra acquired in the presence of 0.0625 mg/mL MCUb-NTD. The solid line is the fit of the net fluorescence emission intensity at 480 nm (black circles) to a one-site binding model that accounts for MCUb-NTD concentration. (C) Comparison of the mean peak ANS fluorescence intensities extracted from the spectra shown in A) (black). The expected intensities due to both dilution and the change in saturation following dilution relative to the starting intensity (pre-dilution) are shown for reference (red). (D) ANS fluorescence emission spectra in the presence of 0.25 mg/mL protein without (black) and with 5 mM CaCl_2_ (red). (E) ANS fluorescence emission spectra in the presence of 0.125 mg/mL protein without (black) and with 5 mM CaCl_2_ (red). (F) Comparison of the mean peak ANS fluorescence intensities extracted from the spectra shown in (D and E). (G) ANS fluorescence emission spectra in the presence of 0.25 mg/mL protein without (black) and with 5 mM MgCl_2_ (red). (H) ANS fluorescence emission spectra in the presence of 0.125 mg/mL protein without (black) and with 5 mM MgCl_2_ (red). (I) Comparison of the mean peak ANS fluorescence intensities extracted from the spectra shown in (G and H). In (D, E, G, and H) ANS concentration was 50 μM, and protein-free ANS emission spectra without (blue) and with 5 mM MgCl_2_ or 5 mM CaCl_2_ (gray) are shown for reference. All experiments were carried out in 20 mM Tris (pH 8.5), 150 mM NaCl, 1 mM DTT at 22.5°C. Data reported are a mean ± SEM of *n* = 3 for all experiments. Statistical analyses were paired t-tests (∗*p* < 0.05, ∗∗*p* < 0.01).

We next evaluated MCUb-NTD solvent exposed hydrophobicity by monitoring 8-anilinonaphthalene sulfonate (ANS) binding. The fluorescence intensity of 50 μM ANS was higher and blue-shifted in the presence of MCUb-NTD at 0.5 mg mL^−1^, 0.25 mg mL^−1^, 0.125 mg mL^−1^, and 0.0625 mg mL^−1^ compared to the buffer alone, consistent with ANS binding to solvent exposed hydrophobic regions^42^ (Figure 3A). A titration of increasing ANS concentrations into 0.0625 mg mL^−1^ MCUb-NTD revealed an apparent equilibrium dissociation constant (K_D_) of 1.5 ± 0.6 μM (Figure 3B). Next, a series of ANS emission spectra in the presence of MCUb-NTD were acquired as a function of doubling dilutions with ANS-free buffer. These dilutions maintained a constant ANS:MCUb-NTD molar ratio. Given the 0.5× ANS concentration at each dilution step and change in binding saturation based our measured K_D_, 54.2%, 56.0% and 58.1% decreases in fluorescence intensity were expected as the protein was diluted from 0.5 mg mL^−1^ to 0.25 mg mL^−1^, 0.125 mg mL^−1^ and 0.0625 mg mL^−1^, respectively. In contrast, less ANS fluorescence than expected solely due to the dilution was observed after each dilution step (Figure 3C), consistent with higher protein concentrations leading to structural changes associated with increased solvent exposed hydrophobicity. Additionally, ANS fluorescence emission spectra in the presence of 0.25 mg mL^−1^ or 0.125 mg mL^−1^ MCUb-NTD showed significantly higher fluorescence intensities when 5 mM CaCl_2_ was added to the samples (Figures 3D–3F). Similarly, 5 mM MgCl_2_ increased ANS binding and fluorescence emission at 0.25 mg mL^−1^ MCUb-NTD (Figures 3G–3I). Collectively, the ANS data indicate that higher protein concentration and divalent cations lead to increased relative solvent exposed hydrophobicity.

### The MCUb and MCU NTDs form a heterodimer resistant to divalent cations

**Figure 4:**
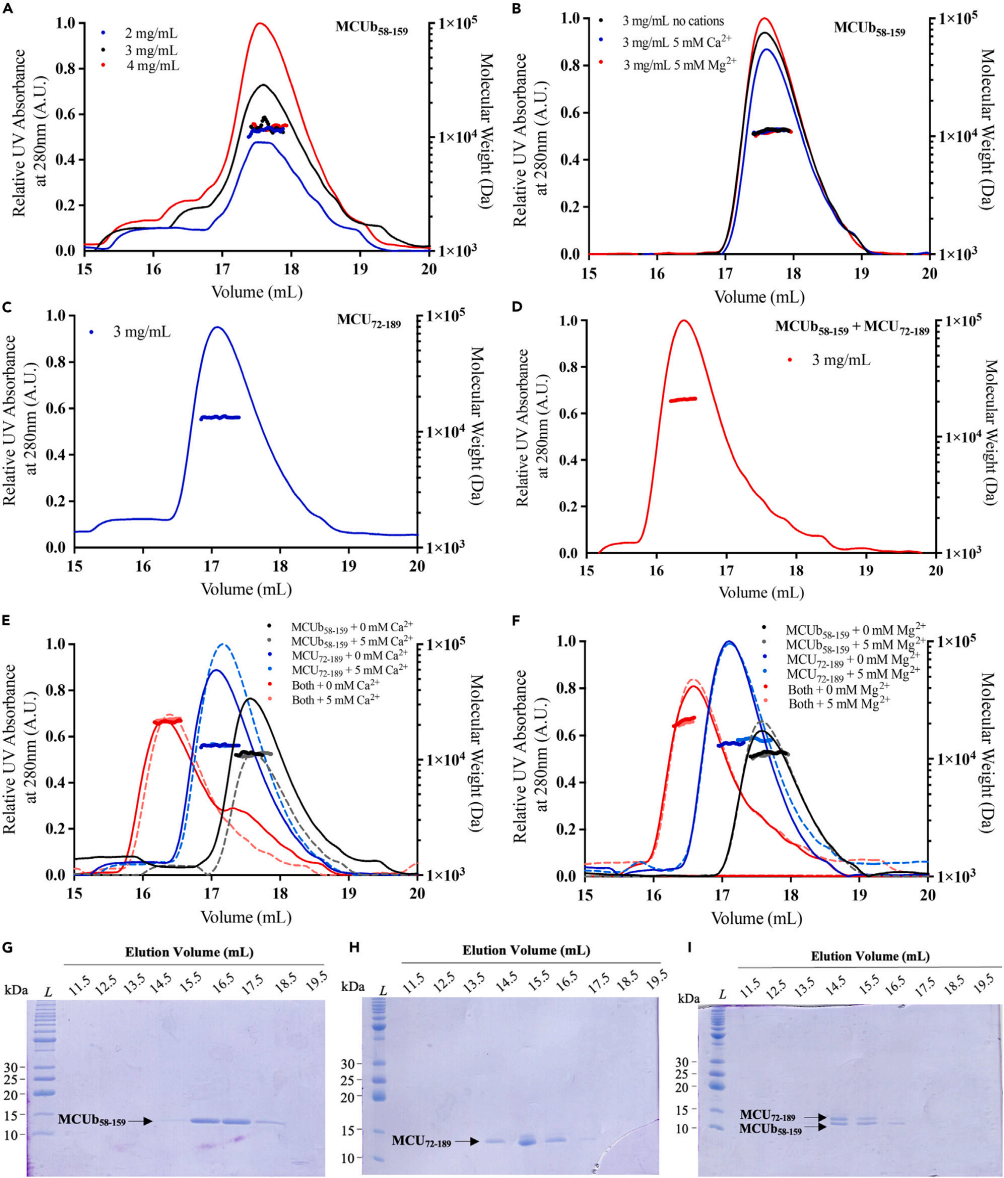
Quaternary structure of MCU- and MCUb-NTDs as a function of protein and divalent cation concentration (A) MCUb-NTD SEC elution profile and MALS-determined molecular weight of particles in the single dominant peak at 2 mg mL^−1^ (blue), 3 mg mL^−1^ (black) and 4 mg mL^−1^ (red) injection concentrations. (B) MCUb-NTD SEC elution profile and MALS-determined molecular weight of particles in the single dominant peak at 3 mg mL^−1^ in the absence (black) and presence of 5 mM CaCl_2_ (blue) and 5 mM MgCl_2_ (red). (C) MCU-NTD SEC elution profile and MALS-determined molecular weight of particles in the single dominant peak at 3 mg mL^−1^ injection concentration. (D) SEC elution profile and MALS-determined molecular weight of a mixed 3 mg mL^−1^ MCUb-NTD and 3 mg mL^−1^ MCU-NTD sample co-eluting in a single dominant peak. (E) SEC elution profiles and MALS-determined molecular weights of individual 3 mg mL^−1^ MCUb-NTD (black), 3 mg mL^−1^ MCU-NTD (blue) and mixed 3 mg mL^−1^ MCUb-NTD +3 mg mL^−1^ MCU-NTD (red), in the absence (solid lines) and presence of 5 mM CaCl_2_ (dashed lines). (F) SEC elution profiles and MALS-determined molecular weights of individual 3 mg mL^−1^ MCUb-NTD (black), 3 mg mL^−1^ MCU-NTD (blue) and mixed 3 mg mL^−1^ MCUb-NTD +3 mg mL^−1^ MCU-NTD (red), in the absence (solid lines) and presence of 5 mM MgCl_2_ (dashed lines). In (A–F), UV absorbance at 280 nm in arbitrary units (A.U.) was used to monitor the elution profile (lines), and the MALS-determined molecular weights are shown under the major peaks (circles). All data were collected using 100 μL protein injections onto a Superdex S200 10/300 GL Increase column in 20 mM Tris (pH 8.5), 150 mM NaCl, 1 mM DTT at 10°C. (G) Elution fractions from MCUb-NTD injected at 3 mg mL^−1^. (H) Elution fractions from MCU-NTD injected at 3 mg mL^−1^. (I) Elution fractions from a MCUb-NTD and MCU-NTD mixed sample injected at a final concentration of 1.5 mg mL^−1^ for each component. In (G–I), ‘L’ denotes the protein ladder. See also Figure S1 and Table S2.

Given the self-association observed here for the MCUb-NTD, the self-association previously observed for the MCU-NTD^33^ and the homology between the domains (Figure 1B), we next used size exclusion chromatography with in-line multi-angle light scattering (SEC-MALS) to assess if these paralogous NTDs interact. First, SEC-MALS was performed on MCUb-NTD, which has a theoretical monomer mass of 12.0098 kDa. At injection concentrations of 2 mg mL^−1^, 3 mg mL^−1^, and 4 mg mL^−1^, MCUb-NTD consistently eluted in a single major peak with MALS-determined molecular weights of 11.4 ± 0.1 kDa, 11.6 ± 0.3 kDa, and 12.2 ± 0.1 kDa, respectively (Figure 4A; Table S2). These data contrast the DLS observations showing higher order oligomerization likely due to the ∼20× dilution that occurs on the SEC column. The presence of 5 mM CaCl_2_ or 5 mM MgCl_2_ in the SEC-MALS experiments did not appreciably alter the elution volume or molecular weights (Figure 4B; Table S2). The theoretical monomeric mass of the MCU-NTD is 13.8637 kDa; SEC-MALS showed that an injection concentration of 3 mg mL^−1^ and after the column dilution, MCU-NTD also exists as a monomer with a measured molecular weight of 13.6 ± 0.2 kDa (Figure 4C; Table S2). Excitingly, when mixed in a ∼1:1 M ratio, the domains formed a heterodimer with a MALS-determined molecular weight of 21.7 ± 0.4 kDa (Figure 4D; Table S2). Further, the mixed sample eluted in a single peak and at an earlier elution volume compared to either of the proteins in isolation. Interestingly, the MCU-NTD:MCUb-NTD interaction was stable in the presence of both 5 mM CaCl_2_ and 5 mM MgCl_2_, as the molecular weight and co-elution volume were minimally affected by the divalent cations (i.e., 22.2 ± 0.1 kDa and 20.8 ± 0.1 kDa in the presence of 5 mM CaCl_2_ and 5 mM MgCl_2_, respectively) (Figures 4E and 4F; Table S2). SDS-PAGE gels of the elution fractions revealed single bands for the individual MCU-NTD and MCUb-NTD injections but two bands in the earlier elution fractions for the mixed samples, confirming co-elution (Figures 4G–4I). Since the proteins elute with a bell-shaped concentration profile in the SEC-MALS experiments, fractions taken from the center of the peak will have higher protein concentration and higher SDS-PAGE band intensities than the flanking fractions. Altogether, SEC-MALS shows that under dilute conditions, MCUb-NTD and MCU-NTD primarily exist as monomers; however, these NTDs interact when mixed, forming stable heterodimers that co-elute in gel filtration experiments.

### The MCUb and MCU NTD interaction is favored by increased secondary structure and stability

**Figure 5:**
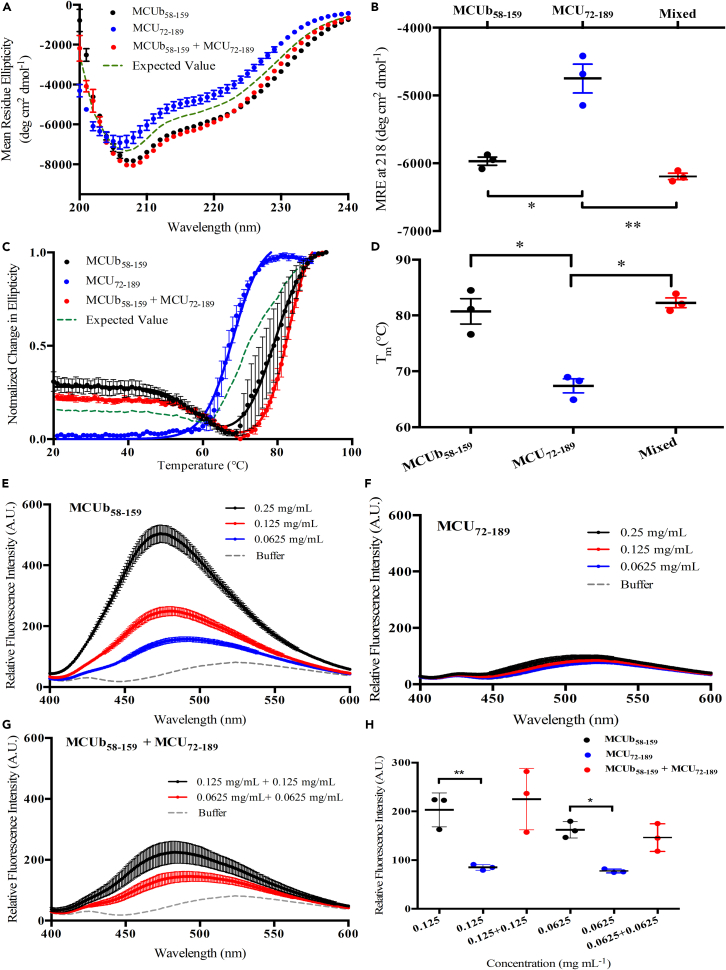
Secondary structure, thermal stability and ANS binding of MCUb-NTD, MCU-NTD and mixed MCUb-NTD + MCU-NTD samples (A) Far-UV CD spectra of MCUb-NTD at 0.5 mg mL^−1^ (black circles), MCU-NTD at 0.5 mg mL^−1^ (blue circles) and a mixed 0.5 mg mL^−1^ MCUb-NTD +0.5 mg mL^−1^ MCU-NTD sample (red circles) at 4°C. The expected MRE for the mixed sample if the interaction did not impact secondary structure is highlighted as a green dashed line. (B) Comparison of the MRE signal at 218 nm for data shown in (A). (C) Thermal melts of MCUb-NTD at 0.5 mg/mL (black circles), MCU-NTD at 0.5 mg/mL (blue circles) and a mixed 0.5 mg mL^−1^ MCUb-NTD +0.5 mg mL^−1^ MCU-NTD sample (red circles). The expected normalized change in ellipticity for the mixed sample if the interaction did not impact thermal stability is highlighted as a green dashed line. (D) Comparison of the T_m_ values extracted from the data shown in (C). Thermal melts were constructed from the change in CD signal at 218 nm as a function of temperature, and solid lines are one or two transition Boltzmann equation fits to the data. (E) ANS fluorescence emission spectra in the presence of 0.25 mg/mL (black), 0.125 mg/mL (red), and 0.0625 mg/mL (blue) MCUb-NTD. (F) ANS fluorescence emission spectra in the presence of 0.25 mg/mL (black), 0.125 mg/mL (red), and 0.0625 mg/mL (blue) MCU-NTD. (G) ANS fluorescence emission spectra in the presence of 0.125 mg/mL MCUb-NTD +0.125 mg/mL MCU-NTD (black) and 0.0625 mg/mL MCUb-NTD +0.0625 mg/mL MCU-NTD (red). (H) Comparison of the mean peak ANS fluorescence intensities extracted from the spectra shown in (E, F, and G). In (E–G), each sample contained 50 μM ANS, and buffer spectra in the absence of protein are shown for reference (gray). All experiments were carried out in 20 mM Tris (pH 8.5), 150 mM NaCl, 1 mM DTT at 22.5°C. Data are means ± SEM of *n* = 3 experiments. Statistical analyses were one-way ANOVA followed by Tukey’s post-hoc test (∗*p* < 0.05, ∗∗*p* < 0.01). See also Figure S2 and Table S1.

We next assessed whether the level of secondary structure and thermal stability of the proteins differed in complex compared to in isolation using far-UV CD spectroscopy. We found that the mixed samples showed significantly more negative MRE compared to MCU-NTD and slightly more negative MRE compared to MCUb-NTD in isolation (Figures 5A and 5B; Table S1). The enhanced negative MRE at ∼208 nm and ∼218 nm compared to the concentration adjusted expected sum of the individual spectra suggests the interaction promotes increased α-helicity and/or β-sheet in MCUb-NTD, MCU-NTD or both. Interestingly, the MCUb-NTD:MCU-NTD sample mixture unfolded with a single transition rather than the stepwise pattern that would be indicative of each protein unfolding independently (Figure 5C). Further, the T_m_ of the single unfolding transition was significantly higher than MCU-NTD (∼15°C) and slightly higher than MCUb-NTD (∼4°C) in isolation (Figures 5C and 5D; Table S1). Thus, MCU and MCUb NTD interactions promote increased folded secondary structure and enhanced thermal stability.

### The MCUb-NTD and MCU NTD exhibit large differences in solvent exposed hydrophobicity

Having observed altered structure and stability in the complex, we next evaluated whether the solvent exposed hydrophobicity of the interacting domains are different than in isolation using ANS binding. Whereas ANS showed high and blue shifted fluorescence emission intensity in the presence of MCUb-NTD at 0.25, 0.125 and 0.0625 mg mL^−1^ (Figure 5E), the ANS fluorescence emission spectra in the presence of MCU-NTD at the same three protein concentrations were similar to the spectrum acquired in the absence of protein (Figure 5F). Thus, MCU-NTD adopts much less solvent exposed hydrophobicity compared to MCUb-NTD under similar solution conditions, despite the high sequence similarity. ANS binding and fluorescence assessed in the presence of mixed MCUb-NTD:MCU-NTD at a final concentration of 0.125 mg mL^−1^ each (i.e., 0.25 mg mL^−1^ total protein concentration) and 0.0625 mg mL^−1^ each, showed similar intensities as ANS in the presence of MCUb-NTD at 0.125 and 0.0625 mg mL^−1^ alone, respectively (Figures 5G and 5H). Therefore, MCU-NTD and MCUb-NTD have marked differences in solvent exposed hydrophobicity but MCUb and MCU NTD interactions do not significantly alter the overall level of solvent exposed hydrophobicity.

### MCU and MCUb NTD interactions favor a heterodimeric quaternary structure

Given our observations that the MCU and MCUb NTDs interact but have very different solvent exposed hydrophobicity levels, we next used DLS to assess if R_h_ distribution profiles change when the proteins are mixed. The MCUb-NTD showed three R_h_ distributions centered at ∼2 nm, 10 nm and 50 nm, with the greatest contribution to scattering intensity derived from the 10 nm and 50 nm distributions (Figure S2A). The primarily bimodal distribution observed here for MCUb-NTD at 67.5 μM (i.e., 0.81 mg mL^−1^) differs from the unseparated size distribution obtained at 0.5 mg mL^−1^ and 1 mg mL^−1^ (Figure S1C) likely due to distinct buffers and temperatures used in the two experiments. At 67.5 μM, the MCU-NTD showed two R_h_ distributions centered at ∼3 nm and 60 nm, with relatively similar contributions to total light scattering intensity by each distribution (Figure S2A). In contrast, the mixed MCU-NTD:MCUb-NTD sample at a total protein concentration of 67.5 μM showed three R_h_ distributions centered at ∼3.5 nm, 15 nm and 50 nm; however, the ∼15 nm distribution was dominant in contribution to total light scattering intensity (Figure S2A).

Next, we used chemical crosslinking followed by SDS-PAGE to confirm that the hetermomeric NTD interactions affect the oligomerization profile of the proteins. *Bis(sulfosuccinimidyl) suberate* (BS-3) crosslinking of MCUb-NTD at 67.5 μM resulted in a systematic laddering with monomer, dimer, trimer, tetramer and higher order bands apparent (Figure S2B). In contrast, BS-3 treated MCU-NTD at 67.5 μM showed only monomer and dimer bands (Figure S2B). Note that intramolecular crosslinking was evident in the MCU-NTD sample with the appearance of band doublets. Remarkably, the mixed samples (i.e., either 67.5 μM or 33.8 μM of each protein in the mixture) demonstrated a clear preference for crosslinking of a dimeric species (Figure S2B). Quantifying the dimer:monomer intensity ratio by densitometry confirmed a significantly higher value for the mixed sample compared to the sum of the ratios of the isolated proteins (Figure S2C). Collectively, the DLS and crosslinking data reveal that the MCU and MCUb NTDs exhibit a preferred heterodimeric quaternary structure.

### MCU and MCUb NTDs interact with sub-*μ*M affinity

**Figure 6:**
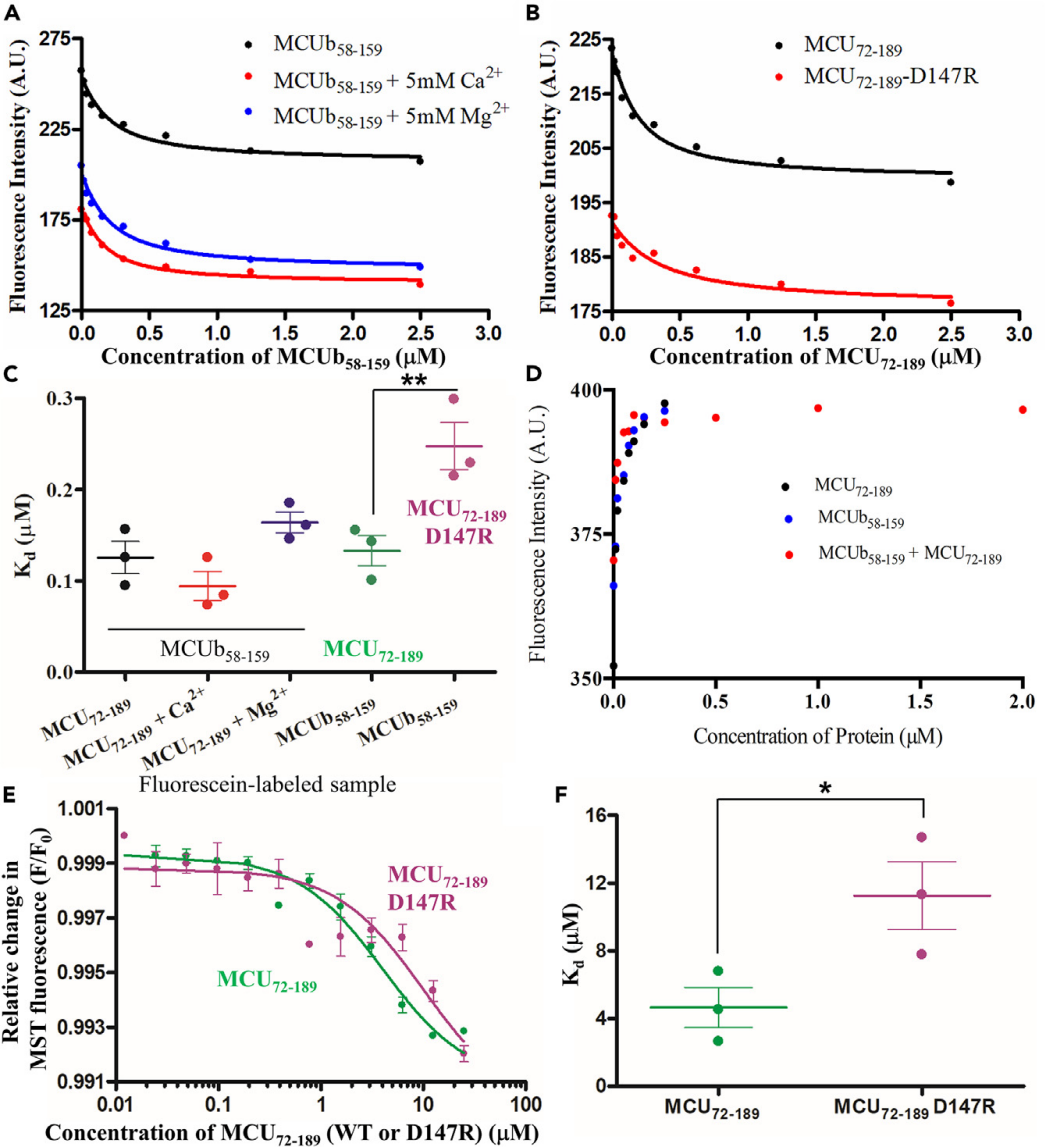
MCUb-NTD:MCU-NTD binding monitored by steady-state fluorescence and microscale thermophoresis (MST) (A) Representative binding curves showing decreases in fluorescein-MCU-NTD fluorescence as a function of increasing MCUb-NTD concentration in the absence (black circles) and presence of 5 mM CaCl_2_ (red circles) or 5 mM MgCl_2_ (blue circles). (B) Representative binding curves showing the decrease in fluorescein-MCUb-NTD fluorescence as a function of increasing MCU-NTD (black circles) and MCU-NTD-D147R (red circles) concentration. (C) Summary of K_D_ values extracted from *n* = 3 separate experiments for each group, using a one site binding model that accounts for fluorescein-labeled protein concentration. K_D_ values are shown from the individual experiments (circles), with means ± SEM indicated. (D) Change in free fluorescein fluorescence as a function of increasing MCU-NTD (black), MCUb-NTD (blue) or mixed MCU-NTD + MCUb-NTD concentrations (red). (E) Averaged MST binding curves showing the relative change in Red-(NHS)-labeled MCUb-NTD MST fluorescence as a function of increasing MCU-NTD (green circles) and MCU-NTD-D147R (pink circles) concentration. Curves are normalized as F/F_0_, where F_0_ is the initial MST fluorescence and F is the MST fluorescence at any timepoint. Data are represented as means ± SEM. (F) Summary of K_D_ values extracted from *n* = 3 separate experiments for each group, using a one site binding model that accounts for labeled protein concentration. K_D_ values are shown from the individual experiment (circles), with means ± SEM indicated. In (A, B and E) solid lines are model fits to the data. (A–D) were carried out with fluorescein-labeled protein or free fluorescein at 0.1 μM in 20 mM Tris (pH 8.5), 150 mM NaCl, 1 mM DTT at 22.5°C, where fluorescein-labeled protein experiments included 0.0001% (v/v) NP-40. (E and F) were completed in 20 mM Tris (pH 8.5), 150 mM NaCl, 1 mM DTT, 0.05% (v/v) NP-40 at 22°C. Statistics in (C and D) are One way ANOVA comparing MCU_72-189_ labeled samples with and without divalent cations and Student’s t test comparing the MCUb_58-159_ labeled samples interacting with WT and D147R MCU_72-189_ (∗*p* < 0.05, ∗∗*p* < 0.01). See also Table S3.

We next used steady-state fluorescence spectroscopy as an initial mode to determine the equilibrium dissociation constant (K_D_) of the interaction. First, we labeled Cys97 of MCU-NTD with fluorescein-methane thiosulfonate (MTS). The MCU-NTD-fluorescein fluorescence intensity systematically decreased with increasing MCUb-NTD concentration, saturating at the highest concentrations (Figure 6A). We also performed the reverse titration after labeling Cys82 and Cys147 of MCUb-NTD with fluorescein and monitoring the decrease MCUb-NTD-fluorescein with increasing MCU-NTD (Figure 6B). Fitting the titration curves to a one site binding model revealed K_D_ values of 125 ± 18 nM for the MCU-NTD-fluorescein experiment and 133 ± 17 nM for the MCUb-NTD-fluorescein titration (Figures 6A–6C; Table S3). The inclusion of 5 mM CaCl_2_ and 5 mM MgCl_2_ in the titrations did not impact the interaction affinity (Figures 6A and 6C; Table S3). Control titrations with free fluorescein revealed a small and systematic increase in the fluorescence, as opposed to the large decrease observed with the labeled proteins (Figure 6D).

Microscale thermophoresis (MST) was used as a second approach to evaluate the K_D_ of the interaction. Red-NHS was covalently linked to primary amines in MCUb-NTD, and differences in temperature-induced fluorescence and molecular thermophoresis as a function of increasing MCU-NTD concentration was monitored. The one-site binding model revealed a K_D_ of 4.65 ± 1.2 μM at 22°C (Figures 6E and 6F; Table S3). The weaker MST-determined affinity compared to our fluorescein-determined measurements is at least in part due to 500× more NP-40 [i.e., 0.05 versus 0.0001% (v/v)], necessary to suppress adhesion to capillaries and experimental variability. Nevertheless, both the MST (acquired with high detergent concentration) and fluorescein measurements suggest that the MCU and MCUb NTDs tightly interact, consistent with the co-elution of the proteins in our SEC.

### MCU and MCUb NTD interactions induce large structural perturbations in both domains

**Figure 7:**
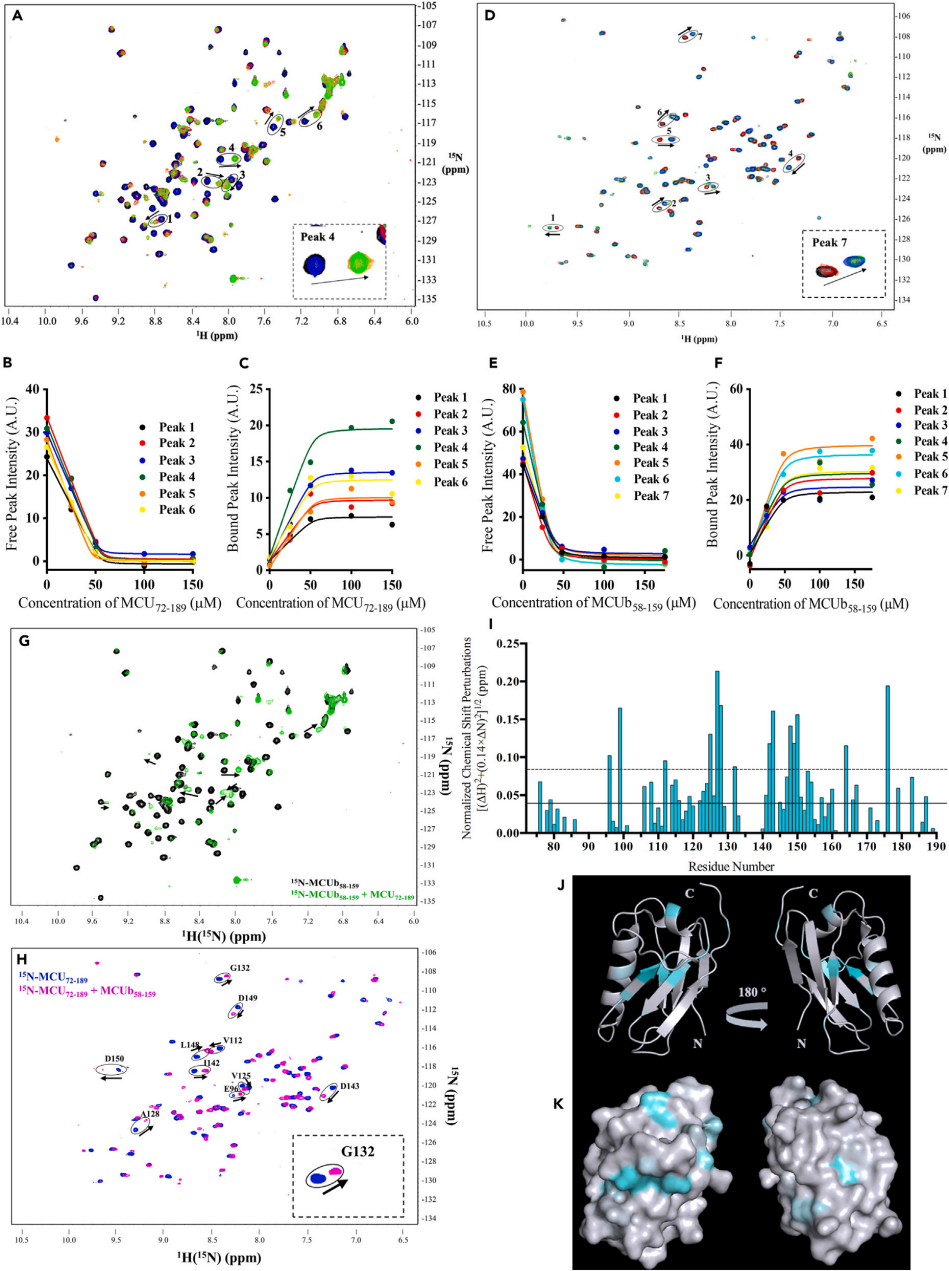
MCUb-NTD:MCU-NTD interactions monitored by solution NMR spectroscopy (A) Overlaid ^1^H-^15^N-HSQC spectra of ^15^N-MCUb-NTD (100 μM) in the absence (black cross peaks) and presence of 25 μM (blue cross peaks), 50 μM (red cross peaks), 100 μM (orange cross peaks), or 150 μM (green cross peaks) MCU-NTD. Amides exhibiting slow exchange were used to estimate the binding affinity from the loss in cross peak intensity of the free state (B) and the gain in cross peak intensity of the bound state (C). (D) Overlaid ^1^H-^15^N-HSQC spectra of ^15^N-MCU-NTD (100 μM) in the absence (black cross peaks) and presence of 25 μM (red cross peaks), 50 μM (orange cross peaks), 100 μM (yellow cross peaks), 150 μM (green cross peaks), or 175 μM (blue cross beaks) MCUb-NTD. Amides exhibiting slow exchange were used to estimate the binding affinity from the loss in cross peak intensity of the free state (E) and the gain in cross peak intensity of the bound state (F). In (A and D), the numbered circles correspond to peak numbers assessed in (B, C, E, and F), respectively, and the arrows highlight the direction of the associated CSP. In (B, C, E, and F) the change in intensity of each ^15^N-MCUb-NTD-derived cross peak and each ^15^N-MCU-NTD-derived cross peak was plotted as a function of increasing MCU-NTD or MCUb-NTD concentration, respectively. Datasets were fit to a one-site binding model taking into account ^15^N-MCUb-NTD or ^15^N-MCU-NTD concentration. K_D_ was a globally shared fitting parameter. Spectra were acquired at 600 MHz in 20 mM HEPES (pH 8.4), 150 mM KCl, 1 mM DTT at 20°C for (A) and 35°C with 1 mM CHAPS for (D). (G) Overlay of free (black cross peaks) and saturated (150 μM MCU-NTD; green cross peaks) ^15^N-MCUb-NTD ^1^H-^15^N-HSQC spectra. Arrows highlight CSP movements in all directions of the spectrum. (H) Overlay of free (blue cross peaks) and saturated (150 mM MCUb-NTD; magenta cross peaks) ^15^N-MCU-NTD ^1^H-^15^N-HSQC spectra. The circles and associated residue numbers indicate the amides undergoing large CSPs (i.e., > mean + 1 × SD), and the arrows highlight the direction of the associated CSP. Spectra were acquired at 600 MHz in 20 mM HEPES (pH 8.4), 150 mM KCl, 1 mM DTT at 20°C in (G) and at 35°C with 1 mM CHAPS in (H). (I) Normalized total ^1^H(^15^N) CSPs caused by saturating interactions of MCUb-NTD with ^15^N-MCU-NTD. Total CSPs are plotted relative to the MCU-NTD residue number with the solid and dashed lines indicating the mean and mean +1 × SD CSP values, respectively. (J) Cartoon representation of the backbone trace of MCU-NTD and (K) surface representation of MCU-NTD with the total CSPs plotted as a minimum to maximum gradient from white to cyan, respectively. MCU-NTD atomic coordinates are from PDB: 5KUJ.^33^ Structure figures were rendered using PyMOL, Version 2.4.0, Schrödinger, LLC. See also Table S3.

Having discovered that the MCUb and MCU NTDs tightly interact, we next aimed to evaluate structural changes at the residue level using solution nuclear magnetic resonance (NMR) spectroscopy. We obtained a spectacular ^1^H-^15^N-MCUb-NTD heteronuclear single quantum coherence (HSQC) in 20 mM HEPES (pH 8.4), 150 mM KCl and 1 mM dithiothreitol (DTT) at 20°C, showing 106 of the 107 expected backbone amide [^1^H(^15^N)] cross peaks. An overlay of a ^15^N-MCUb-NTD HSQC spectral series acquired with increasing MCU-NTD concentrations revealed changes in ^1^H(^15^N) position and intensity, consistent with the MCUb-NTD:MCU interaction (Figure 7A). Further, many ^1^H(^15^N) crosspeaks showed decreases in intensity at the initial position concomitant with increases in intensity at a new position as a function of increasing MCU-NTD concentration, indicating slow exchange (Figure 7A). Simultaneous fitting of the loss in intensity of six ^1^H(^15^N) cross peaks, chosen due to easily inferred bound state positions, revealed a K_D_ of 180 ± 129 nM (Figure 7B; Table S3). Similarly, simultaneously fitting the gain in intensity of the bound state positions showed a K_D_ of 737 ± 971 nM (Figure 7C; Table S3). A reverse titration was performed using ^15^N-MCU-NTD and increasing concentrations of MCUb-NTD. This titration was set up with solution conditions optimized for the ^15^N-MCU-NTD spectrum [i.e., 20 mM HEPES (pH 8.4), 150 mM KCl, 1 mM DTT, 1 mM CHAPS at 35°C]. An overlay of the ^1^H-^15^N-HSQC spectra revealed several peaks undergoing a similar slow exchange as observed in the ^15^N-MCUb-NTD titration (Figure 7D). Simultaneous fitting of the loss in intensity of seven ^1^H(^15^N) cross peaks revealed a K_D_ of 150 ± 262 nM (Figure 7E; Table S3), with the gain in intensity of the bound state positions showing a K_D_ of 1,249 ± 1,318 nM (Figure 7F; Table S3).

Both the ^15^N-MCUb-NTD and ^15^N-MCU-NTD HSQC spectra exhibited widespread changes in ^1^H(^15^N) crosspeak position and intensity, indicating global structural changes in both proteins upon interaction (Figures 7G and 7H). While the backbone atom chemical shifts for MCUb-NTD are unknown, many of the MCU-NTD ^1^H(^15^N) chemical shifts have been assigned. Plotting the magnitude of the ^1^H(^15^N) chemical shift perturbations (CSPs) relative to the MCU-NTD sequence revealed many of the changes greater than the mean +1 × standard deviation (SD) are localized on the β-sheets of the domain. Specifically, residues in β3, β4 and β5 strands show large CSPs. Further, large perturbations are observed for residues D147 and D148, two of three acidic residues that comprise the MCU regulating acidic patch (MRAP)^33^ (Figure 7I). Plotting the CSP magnitudes as a gradient on the backbone ribbon and surface representations of the MCU-NTD structure shows the largest CSPs primarily localized to one face of the domain, where large β4, β5, α1 and α2 CSPs are adjacent to each other (Figures 7J and 7K). Nevertheless, consistent with the widespread CSPs, larger perturbations are present on the opposite face of the protein at β3, which is also adjacent to both α-helices (Figures 7J and 7K). Collectively, our NMR data confirm a slow exchange, tight binding interaction between the MCUb and MCU NTDs and reveal changes in the chemical environments of most ^1^H(^15^N) on both domains.

### Deletion of the MCUb-NTD impairs MCUb and MCU co-localization in mammalian cells

**Figure 8:**
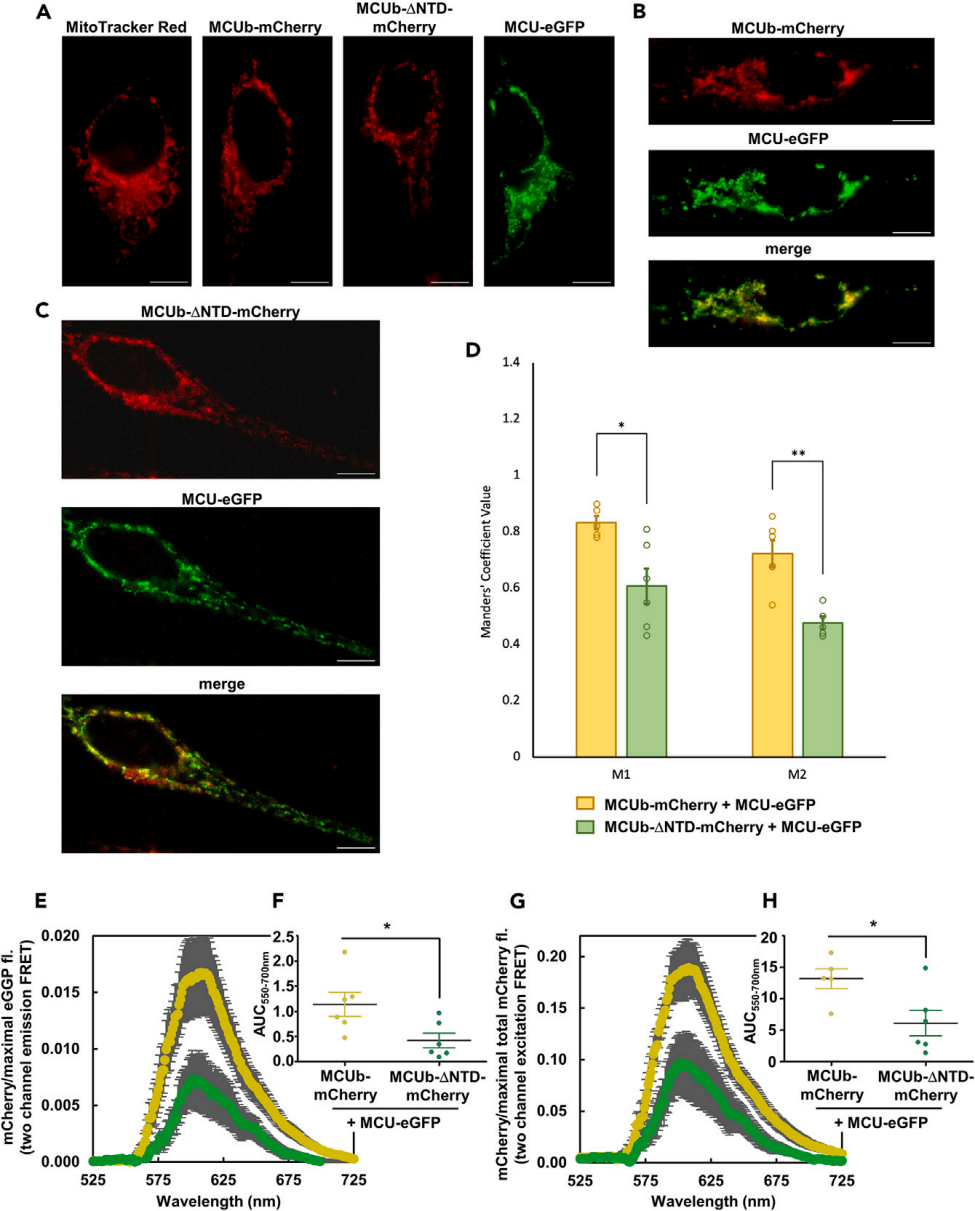
NTD-dependent co-localization of MCU and MCUb in HeLa cells (A) Localization of separately transfected MCUb-mCherry (red), MCU-eGFP (green) and MCUb-ΔNTD-mCherry (red) compared to MitoTracker red (red) in fixed HeLa cells. (B) Localization of co-transfected MCUb-mCherry (red) and MCU-eGFP (green) in fixed HeLa cells. The merged green and red channels (merge) highlights regions of co-localization (yellow). (C) Localization of co-transfected MCUb-ΔNTD-mCherry (red) and MCU-eGFP (green) in fixed HeLa cells. The merged green and red channels (merge) highlights regions of co-localization (yellow). (D) Manders’ M1 and M2 coefficients assessing the co-occurrence of red to green pixels and green to red pixels, respectively, for both the co-transfected MCU-eGFP + MCUb-mCherry (yellow) and MCU-eGFP + MCUb-ΔNTD-mCherry (green) cells. (E) Two channel emission eGFP:mCherry FRET spectra of HeLa cells co-transfected with MCU-eGFP + MCUb-mCherry (yellow) and MCU-eGFP + MCUb- ΔNTD-mCherry (green). (F) Area under the curve (AUC; 550–700 nm) comparison of the two channel emission FRET spectra. (G) Two channel excitation mCherry:total mCherry FRET spectra of HeLa cells co-transfected with MCU-eGFP + MCUb-mCherry (yellow) and MCU-eGFP + MCUb-ΔNTD-mCherry (green). (H) AUC (550–700 nm) comparison of the two channel excitation FRET spectra. White scale bars in (A–C) represent 10 μm. Data in (D) are expressed as means ± SEM from n = 5–6 cells co-expressing MCUb-mCherry or MCUb-ΔNTD-mCherry and MCU-eGFP from 2 to 3 separate co-tranfections. Data in (E–H) are means ± SEM from n = 5–6 separate co-transfections. Statistical analyses were unpaired t-tests (∗*p* < 0.05, ∗∗*p* < 0.002). See also Figure S3.

Having found that the isolated NTDs tightly interact *in vitro*, we next assessed the role of this interaction on MCU:MCUb co-localization *in cellulo*. We co-transfected either a full-length human MCUb-mCherry fusion construct or MCUb-mCherry with NTD residues 79–160 deleted (MCUb-ΔNTD-mCherry) with full-length MCU-eGFP in HeLa cells. MCUb-mCherry, MCUb-ΔNTD-mCherry and MCU-eGFP showed a similar fluorescence distribution as MitoTracker Red, consistent with the mitochondrial localization of all constructs (Figure 8A). To assess mCherry and eGFP co-localization, we determined Manders’ coefficients. Cells co-expressing MCU-eGFP and MCUb-mCherry showed a high co-occurrence of red to green pixels (M1; 0.83 ± 0.02) as well as green to red pixels (M2; 0.72 ± 0.05), consistent with strong co-localization of these wild-type protein constructs (Figures 8B and 8D). In contrast, cells co-expressing MCU-eGFP with MCUb-ΔNTD-mCherry showed significantly lower M1 and M2 coefficients of 0.61 ± 0.06 and 0.48 ± 0.02, respectively, indicative of disrupted MCU:MCUb co-localization upon NTD deletion from MCUb (Figures 8C and 8D). We also assessed co-localization using semi-quantitative FRET measurements. When exciting eGFP (donor), mCherry fluorescence emission (acceptor) from HeLa cells co-expressing MCU-eGFP and MCUb-ΔNTD-mCherry showed reduced intensity compared to cells co-expressing MCU-eGFP and MCUb-mCherry, normalized using the maximum eGFP signal (i.e., two channel FRET emission ratios) (Figures 8E and 8F). Similarly, when exciting eGFP, mCherry fluorescence emission intensity was significantly lower for MCU-eGFP and MCU-ΔNTD-mCherry co-expressing cells compared to cells co-expressing MCU-eGFP and MCUb-mCherry, normalized using total mCherry fluorescence after separate excitation of mCherry (i.e., two channel FRET excitation ratios) (Figures 8G and 8H). Collectively, both the Manders green/red pixel co-occurrence and FRET analyses indicate reduced co-localization of MCU and MCUb upon deletion of the MCUb-NTD.

Given that MRAP residues exhibited some of the largest CSPs upon interacting with MCUb-NTD (Figure 7I), we next tested whether MCU MRAP mutations affect co-localization with MCUb-mCherry. We previously showed MCU-D131R-GFP and MCU-D147R-GFP are mitochondrially localized^33^ and similarly found a mitochondrial-like distribution here when co-expressed with MCUb-mCherry in HeLa cells (Figures S3A–S3C). However, the MCUb-mCherry pixel co-occurrence with MCU-D131R-eGFP was significantly lower (M1; 0.42 ± 0.05) compared to the MCU-eGFP (M1; 0.88 ± 0.02) or MCU-D147R-eGFP (M1; 0.79 ± 0.04) expressing cells, suggesting weaker co-localization of MCUb-mCherry with the MCU-D131R-eGFP mutant (Figure S3D). We^33^ and others^34^ previously showed that mutations at D131 or D147 inhibit mitochondrial Ca^2+^ uptake and so did not repeat the mitochondrial Ca^2+^ uptake experiments with these mutants here. However, given that D147 within MRAP was specifically identified to undergo large CSPs, we assessed the effects of the D147R mutation in our *in vitro* binding experiments. Consistent with MRAP playing a role in the MCUb-NTD:MCU-NTD interaction, the MCU-NTD-D147R showed ∼2-fold increased equilibrium dissociation constants measured by steady state fluorescein and MST titrations, although the interaction remained tight overall (Figures 6C and 6F; Table S3), consistent with no change in co-localization (Figure S3).

### Deletion of the MCUb-NTD restores mitochondrial Ca^2+^ uptake in mammalian cells

**Figure 9:**
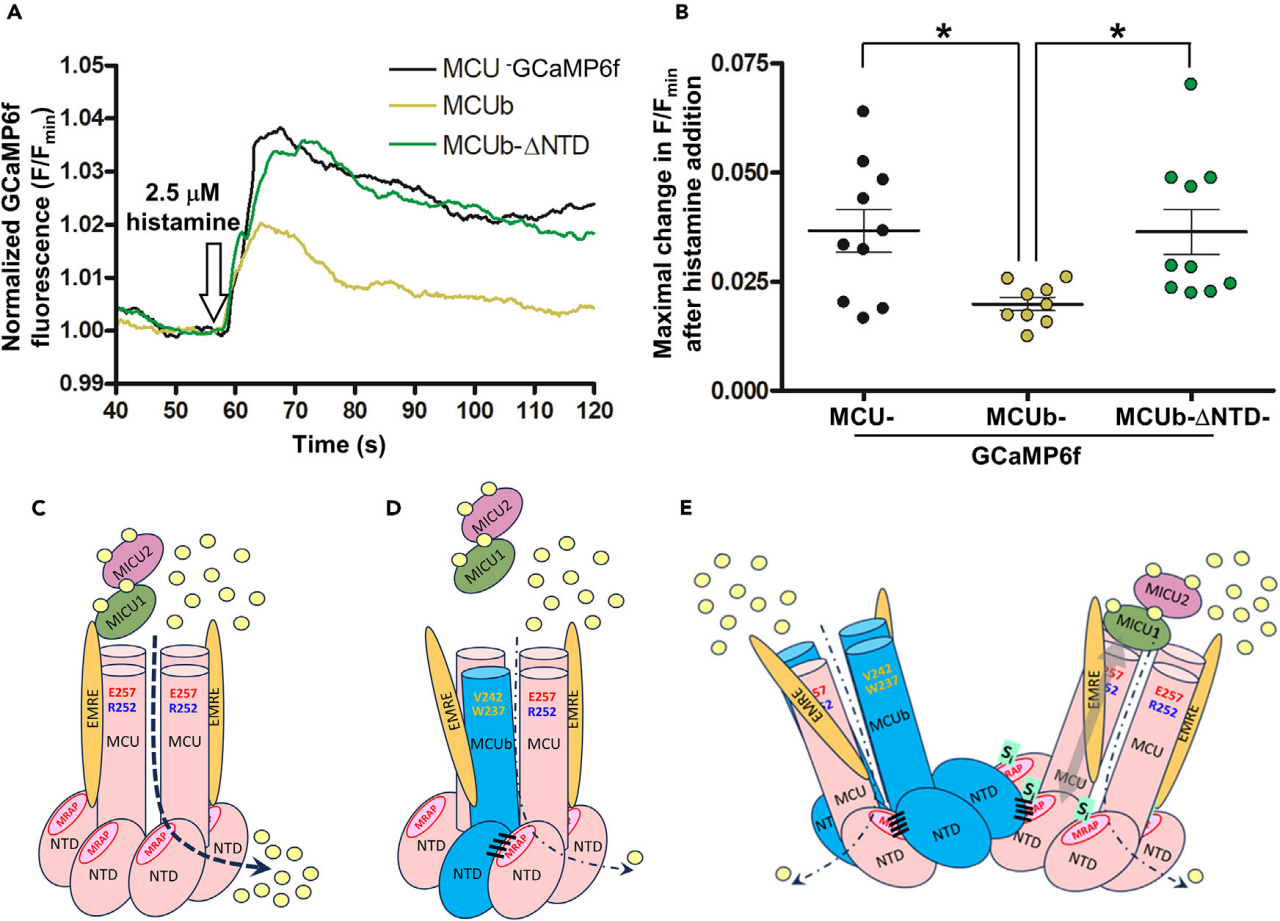
Role of the MCUb-NTD in regulating mitochondrial Ca^2+^ uptake (A) Representative GCaMP6f fluorescence traces reporting relative changes in mitochondrial Ca^2+^ before and after 2 mM CaCl_2_/2.5 μM histamine-containing HBSS perfusion. Data were normalized as F/F_min_, where F is fluorescence at any time point and F_min_ is the minimum fluorescence intensity prior to histamine addition. (B) One-way ANOVA followed by Tukey’s post-hoc test comparing maximal histamine-induced fluorescence changes, where ∗*p* < 0.05. Maximal in F/F_min_ were calculated by subtracting the averaged F/F_min_ baseline pre-histamine addition from the maximum F/F_min_ post-histamine addition. (C) Homotetrameric MCU (pink) channel within one mtCU showing high Ca^2+^ conductance under high IMS Ca^2+^ conditions. (D) Heterotetrameric MCU (pink):MCUb (blue) channel within one mtCU depicting low Ca^2+^ conductance under high IMS Ca^2+^ conditions. (E) Heterotetrameric MCU:MCUb channel within one mtCU interacting with a homotetrameric MCU channel within a second mtCU depicting low Ca^2+^ conductance for both complexes under high IMS Ca^2+^ conditions. In (C–E), the MCU regulating acidic patch (MRAP) within the MCU-NTDs (pink), EMRE (orange) at less than 1×EMRE:1×MCU/MCUb stoichiometry and MICU1 (green)/MICU2 (magenta) on the IMS side of the channel are indicated. The thick and thin dashed lines depict high and low Ca^2+^ conductance, respectively, and the yellow spheres represent Ca^2+^. In (D–E), mechanisms contributing to low MCUb-mediated Ca^2+^ conductance include critical residue substitutions within the TM region (i.e., R252 and E257 within MCU existing as W237 and V242 in MCUb),^21^^,^^28^^,^^29^ altered EMRE interactions that disrupt MICU1/MICU2 gatekeeping interactions with mtCU,^27^^,^^28^^,^^29^^,^^43^ changes in the pore architecture of mtCU with MCUb components^28^^,^^32^ and interactions of a heterotetrameric MCU/MCUb channel within one mtCU with a homotetrameric MCU channel within a second mtCU via the NTDs, analogous to the V-shaped mtCU dimers observed in cryo EM structures.^11^^,^^44^^,^^45^^,^^46^ Assembly of heterotetrameric MCU/MCUb channels within one mtCU and in the V-shaped mtCU dimers is promoted by the ∼150-330× increased MCUb-NTD:MCU-NTD affinity. Tight MCUb-NTD interactions with the MRAP region of MCU-NTD (black bars) across mtCU complexes could allosterically couple through the TMs to MICU1/MICU2 and lead to inhibition in homotetrameric MCU mtCU complexes (gray double headed arrow). This MCU-NTD-MICU1/MICU2 coupled inhibitory mechanism has been previously shown upon Ca^2+^ binding to the inhibitory site (S_i_) within MRAP.^34^

To link the suppressed co-localization of MCUb-ΔNTD and MCU with changes in mitochondrial Ca^2+^ uptake, we replaced the mCherry fluorescent protein on MCUb and eGFP on MCU with a GCaMP6f Ca^2+^ sensor, previously used to directly assess matrix Ca^2+ 42^. To ensure matrix Ca^2+^ was monitored at sites of MCUb or MCUb-ΔNTD localization with the highest sensitivity, we used this fusion approach. Note that MCU-GFP fusions show no differences in localization compared to endogenous MCU.^36^ We performed histamine-induced mitochondrial Ca^2+^ uptake assays in intact HeLa cells after overexpression of MCU-GCaMP6f, MCUb-GCaMP6f or MCUb-ΔNTD-GCaMP6f. A minimum of 24 h post-transfection, cells were perfused with Ca^2+^-free HEPES buffered saline solution (HBSS) and after a 60 s baseline fluorescence collection focused on GCaMP6f positive cells only, cells were perfused with complete HBSS supplemented with 2.5 μM histamine. The data show that HeLa cells over-expressing MCUb-ΔNTD-GCaMP6f permit significantly more histamine-induced mitochondrial Ca^2+^ uptake than MCUb-GCaMP6f expressing cells (Figures 9A and 9B). Note that the similar uptake levels observed for MCUb-ΔNTD-GCaMP6f and MCU-GCaMP6f expressing cells does not imply that Ca^2+^ permeation is enhanced to MCU-like levels upon NTD deletion; rather, this observation could be due to enhanced activity of the endogenous MCU complexes in the presence of MCUb-ΔNTD or suppressed MCU-GCaMP6f activity due to the nature of the fusion, insufficient regulators to maximally activate the overexpressed MCU-GCaMP6f or a combination of these factors.

## Discussion

The MCU-NTD can both positively and negatively regulate mtCU through divalent cation binding and post-translational modification sites.^33^^,^^34^^,^^35^^,^^36^^,^^37^^,^^38^^,^^39^ In contrast, the role of the MCUb-NTD in mtCU function remains largely enigmatic. Thus, we here characterized the biophysical, structural and functional nature of the human MCUb-NTD to better understand how MCUb regulates the channel despite the high sequence similarity to MCU (Figure 1).

Our spectroscopic data revealed a strong MCUb-NTD concentration-dependent level of structure and stability, which has direct functional implications given the distinct tissue-specific expression patterns observed for MCUb. MCUb expression is high in lung, heart, brain, hematopoietic and immune cells, but much lower in skeletal muscle, showing a reciprocal relationship with MCU expression.^21^^,^^29^ We found that lower MCUb-NTD concentrations favor increased secondary structure and stability (Figure 2), contrasting the behavior of MCU-NTD. Higher protein concentration typically increases thermodynamic stability of homo-oligomerizing proteins,^47^ and MCU-NTD adheres to this principle. The peculiar concentration/stability behavior of MCUb-NTD would favor stability in tissues of low MCUb expression. We also found that MCUb-NTD shows much higher levels of solvent-exposed hydrophobicity/ANS binding compared to MCU-NTD (Figure 3). The high level of solvent exposed hydrophobicity in MCUb-NTD most likely underlies the observed tendency for higher-order homo-oligomerization (Figure S1) and heteromeric interactions.

Interestingly, Mg^2+^ and Ca^2+^ interactions with MCUb-NTD also cause a destabilization, although MgCl_2_ treatment causes more instability than CaCl_2_ (Figure 2H). In the absence of high-resolution MCUb-NTD structures with bound Ca^2+^ and Mg^2+^, it is difficult to pinpoint the precise interaction sites of the divalent ions. Since the MCU-NTD MRAP residues (i.e., D131, D147, D148) are not fully conserved in MCUb-NTD (i.e., D116, N132, D133), we believe the interaction sites of Ca^2+^ and Mg^2+^ on MCUb-NTD are distinct from MCU-NTD.^33^ Further, Mg^2+^ binds more tightly to water, has a larger solvated radius, longer solvated lifetime and greater solvated rigidity than Ca^2+^,^48^^,^^49^ all factors that could contribute to disparate interaction sites and destabilization effects. Given increased protein concentration and divalent cation binding are both destabilizing, it is possible the MCUb-NTD conformations adopted upon homodimerization and cation binding are similar. Consistent with this notion, at our highest MCUb-NTD protein concentration (2 mg/mL), we observed a clear bimodal distribution of hydrodynamic sizes centered at ∼10 nm (small oligomers) and ∼50 nm (large oligomers), and a remarkably similar bimodal distribution was observed upon Mg^2+^ treatment but at lower protein concentrations (0.5 mg/mL) (Figure S1).

Our SEC-MALS data revealed that MCU-NTD and MCUb-NTD interact, co-eluting despite the ∼20× dilution on the SEC column (Figure 4). The heteromeric interaction observed here is coherent with past modeling and qualitative experiments suggesting these domains interact.^50^ Interestingly, whereas ∼ mM levels Mg^2+^ and Ca^2+^ have been shown to disrupt MCU-NTD interactions, we found the heteromeric interaction remains intact even though these divalent cations destabilize MCUb-NTD (Figure 2) and MCU-NTD.^33^ Congruent with the tight binding evidenced by the column co-elution, steady-state fluorescence, MST and NMR spectroscopy titrations reported K_D_ values in the ∼90–4,650 nM range (Figures 6 and 7; Table S3). Indeed, the NMR titrations revealed many peak positional changes in slow exchange, consistent with tight binding.^51^ Experiment-specific variations in these K_D_s are due to the different labeling approaches, protein concentrations and associated effect on oligomerization, experimental temperatures (i.e., 20°C–35°C) and buffers with and without detergents. Nevertheless, despite variability all our experiments indicate that MCUb-NTD and MCU-NTD tightly interact.

We found the heteromeric interaction is favored by increased secondary structure and stability (Figure 5). Consistent with the binding-induced structure and co-stabilization, we observed large CSPs in the ^1^H-^15^N HSQC spectra of both domains (Figure 7). Remarkably, many of the largest CSPs occurred on residues that make up the MCU-NTD MRAP region. Mutations or divalent cation binding within MRAP shift the self-association equilibrium of the MCU-NTD toward monomer and suppress the assembly^33^ and function of the full-length mtCU channel.^33^^,^^34^ Thus, it is tempting to speculate that binding of the MCUb-NTD to the MCU-NTD may, at least in part, inhibit MCU function by perturbing MRAP similar to mutational or divalent cation binding effects within MRAP. Note that the MCUb-NTD atom-specific chemical shifts are unknown so no CSP mapping was performed.

Consistent with other studies demonstrating an inhibitory effect of MCUb on mitochondrial Ca^2+^ permeation,^21^^,^^27^^,^^43^^,^^52^ we found that HeLa cells overexpressing MCUb show suppressed mitochondrial Ca^2+^ uptake (Figures 9A and 9B). Remarkably, overexpressing MCUb with a deleted NTD (MCUb-ΔNTD) showed significantly higher mitochondrial Ca^2+^ uptake compared to MCUb with an intact NTD. MCUb silencing, knockout (KO) or overexpression does not alter mitochondrial membrane potential in HeLa cells, as assessed by TMRM^21^ and JC-1 fluorescence.^27^ Congruently, we found here that MCUb-ΔNTD overexpression achieved similar maximal mitochondrial Ca^2+^ uptake as MCU overexpressing cells, suggesting that mitochondrial membrane potential is intact with this previously untested construct (see Figure 9B). Previous studies have suggested MCUb-specific decreases in mtCU Ca^2+^ conductance due to *i*) altered pore geometry,^28^^,^^32^ *ii*) key amino acid substitutions in MCUb compared to MCU (i.e., R252W and E257V) that could either contribute to different pore geometry and/or alter surface electrostatics^21^^,^^28^^,^^29^ and *iii*) altered interactions with regulators (i.e., EMRE and MICUs).^27^^,^^28^^,^^29^^,^^43^ Our work reveals that MCU-NTD has a minimum of ∼60–130× greater affinity for interactions with MCUb-NTD (i.e., based on the average of the experimentally determined K_D_s for the wild-type (WT) proteins in the absence of divalent cations; Table S3) compared to self-association (i.e., K_D_ ∼60–130 μM^33^). MCUb-NTD also has a much lower tendency for self-association as evidenced by our SEC-MALS data, which shows no homodimer. MCU KO increases basal endoplasmic reticulum (ER) Ca^2+^, accelerates ER Ca^2+^ refilling and enhances cytosolic Ca^2+^ transients in response to ER Ca^2+^ store depletion^53^; thus, MCUb-dependent decreases in mitochondrial Ca^2+^ uptake could affect cytosolic Ca^2+^ signals.

The nature of the tight heteromeric interactions have implications for both assembly of MCUb within mtCU and mechanism of decreased Ca^2+^ conductance. First, tight MCU-NTD:MCUb-NTD interactions would favor heteromeric assembly of MCUb with MCU over homomeric assembly (Figures 9C and 9D). Indeed, we showed decreased MCU:MCUb co-localization upon deletion of the MCUb-NTD (Figure 8). Second, the MCU-NTD interaction site, as revealed in this work, is localized on the MRAP region, previously shown to decrease conductance upon Ca^2+^ binding.^33^^,^^34^ Thus, this interaction could inhibit Ca^2+^ conductance either within one tetrameric channel and across channels interacting via the NTDs (Figures 9D and 9E) as revealed in cryo-EM structures of dimers of mtCU channels.^11^^,^^44^^,^^45^^,^^46^ This later mechanism would be dependent on the presence of the inhibitory site (S_i_) within MRAP and transmembrane coupling to the MICU1/MICU2 gatekeepers as previously described.^34^ Note that our work does not exclude a role for the transmembrane domain in the assembly of the MCU:MCUb heteromeric channel. While Manders’ coefficients between MCU-eGFP and MCUb-mCherry are significantly decreased upon MCUb-NTD deletion, they remain relatively high ∼0.5–0.7 (Figure 8). Congruently, the TM domains of *Trypanosoma brucei* were shown to be important determinants of MCU paralog interactions within the mtCU.^54^ MICU1 plays an important role in spatially anchoring the MCU complex at inner boundary membranes upon histamine-induced Ca^2+^ elevation.^55^ However, it is unclear whether this mechanism can occur via direct MCUb binding given studies showing no interactions between MCUb and MICU1.^27^^,^^32^

### Limitations of the study

A limitation of the work is our focus on a domain that is taken out of the context of the full-length protein. The MCU-NTD is well-folded and has been crystalized in isolation, with the structure and biophysical properties faithfully informing on the structure and function of full-length MCU.^33^ Here, we show that MCUb-NTD is similarly well folded. Our cell assays use an MCUb-ΔNTD construct with residues 79–160 deleted. Previous work on human MCU-ΔNTD (residues 75–165 deleted) found this protein to fold with the expected levels of secondary structure and formed a Ca^2+^ permeable channel in HeLa cells.^56^ Several other studies have also showed that MCU-ΔNTD constructs from lower and higher order organisms all form functional Ca^2+^ channels.^8^^,^^11^^,^^57^^,^^58^^,^^59^ Given the high sequence similarity between the human MCUb and MCU and the established tolerance of MCU channel function to NTD deletion, we do not expect the MCUb-ΔNTD to be misfolded. Another limitation is the large range of protein concentrations used in the work, necessitated by the specific assay performed. Given the sensitivity of stability on protein concentration observed here, this large range of protein concentrations likely contributed to data variability.

Previous work showed recombinant MCUb reconstituted alone in bilayers can permeate Na^+^, indicating the formation of a functional channel, while the same experimental setup did not show Ca^2+^ permeation.^21^ However, this artificial system was missing all regulating proteins, and so data on the ability of MCUb to form a Ca^2+^ permeable channel remains limited. Given the DIME motif critical for Ca^2+^ selectivity and permeation is conserved in MCUb and the high overall sequence similarly between MCU and MCUb, it is likely MCUb is capable of Ca^2+^ permeation. There is precedence for Ca^2+^ ion channels to express highly similar paralogs with distinct functional properties (i.e., ORAI1, ORAI2 and ORAI3) and the modulation of channel function depending on paralog hetero-oligomer composition.^60^^,^^61^^,^^62^^,^^63^ Overall, we find an extraordinary divergence in the biophysical and structural properties of MCUb-NTD compared to MCUb-NTD despite the high sequence similarity of these regulatory domains. These disparities endow a preference for hetero-oligomerization driven by a remarkable ∼2 orders of magnitude higher affinity compared to homo-oligomerization. Once integrated into mtCU, decreased Ca^2+^ conductance ensues due to perturbed pore geometry, electrostatics, interactions with regulators and MRAP contacts, effects that need not be mutually exclusive.

## STAR★Methods

### Key resources table

**Table:** 

REAGENT or RESOURCE	SOURCE	IDENTIFIER
**Bacterial and virus strains**		

BL21 codon plus *E. coli*	Agilent	Cat# 230280
DH5 alpha *E. coli*	Thermo Fisher	Cat# 18265017

**Chemicals, peptides, and recombinant proteins**		

TRIS	BioShops	Cat# TRS001.5
HEPES	BioShops	Cat# HEP001.250
DTT	BioShops	Cat# DTT001.50
NaCl	BioShops	Cat# SOD002.10
CHAPS	BioShops	Cat# CHA003.10
Bio tryptone	BioShops	Cat# TRP402.205
Yeast extract	BioShops	Cat# YEX401.205
kanamycin	BioShops	Cat# KAN201.25
Urea	BioShops	Cat# URE001.5
Gdn-HCl	BioShops	Cat# GUA003.5
Na2HPO4	BioShops	Cat# SPD307.1
KH2PO4	BioShops	Cat# PPM666.500
D-glucose	BioShops	Cat# GLU501.1
Thiamine	Thermo Fisher	Cat# A1956022
Biotin	Bioshops	Cat# BIO302.5
MgCl2	Bioshops	Cat# MAG520.500
CaCl2	Bioshops	Cat# CCL444.500
Nonidet P-40	BDH	Cat# 56009
Bovine thrombin	MilliporeSigma	Cat# 605157-1KU
IPTG	Wisent	Cat# 800-050-XG
ANS	MilliporeSigma	Cat# A1028-5G
PolyJet	SignaGen	Cat# SL100688
DMEM with high glucose w/High Glucose, L-Glu, Pyruvate; w/o HEPES	Wisent	Cat# 319-005-CL
Trypsin	Wisent	Cat# 325-043-EL
FBS	Wisent	Cat# 095-150
Pen/Strept	Wisent	Cat# 450-201-EL
Paraformaldehyde	EM Sciences	Cat# EMS15710
BS-3	Thermo Fisher	Cat# PI21580
Fluorescein-MTS	Toronto Research Chemicals	Cat# TRC-A609615
^15^N-NH_4_Cl	MilliporeSigma	Cat# 299251-50G
DSS	MilliporeSigma	Cat# 178837
D2O	Cambridge Isotopes Laboratories	Cat# DLM4-100

**Critical commercial assays**		

HisPur Ni-NTA	Thermo Fisher	Cat# 88221
Red-NHS labeling kit	NanoTemper	Cat# MO-L011

**Experimental models: Cell lines**		

HeLa	ATCC	CCL-2

**Recombinant DNA**		

pET28a-MCUb_58-159_	This paper	N/A
pET28a-MCU_72-189_	Lee et al.^33^	N/A
pCMV-MCU-eGFP	Lee et al.^33^	N/A
pCMV-MCU-D131R-eGFP	Lee et al.^33^	N/A
pCMV-MCU-D147R-eGFP	Lee et al.^33^	N/A
pcDNA-MCUb-mCherry	This paper	N/A
pcDNA-MCUb-ΔNTD-mCherry	This paper	N/A
pEGFP-N1	Clonetech	Cat# 6085-1; discontinued
pEGFP-MCU-GCaMP6f	This paper	N/A
pEGFP-MCUb-GCaMP6f	This paper	N/A
pEGFP-MCUb-ΔNTD-GCaMP6f	This paper	N/A
pcDNA3.1+-MCUb-C-(k)DYK	Genescript	Cat# OHu13258
pCMV-Mito4x-GCaMP6f	Ashrafi et al.^64^	Addgene plasmid #127870
pCMV-mCherry-STIM1	Luik et al.^65^	N/A

**Software and algorithms**		

GraphPad Prism v4.02	GraphPad Software, Inc.	https://www.graphpad.com/
nmrPipe v10.9	Delaglio et al.^66^	https://www.ibbr.umd.edu/nmrpipe/install.html
ImageJ2 v2.14.0/1.54f	Rueden et al.^67^	https://imagej.net/software/imagej2/
R-4.2.1	R Project for Statistical Computing	https://www.R-project.org/

### Resource availability

#### Lead contact

Further information and requests for resources and reagents should be directed to and will be fulfilled by the lead contact, Peter B. Stathopulos (pstatho@uwo.ca).

#### Materials availability

Plasmids generated in this study are available upon request to the lead contact.

#### Data and code availability

• All data reported in this will be shared by the lead contact upon request.
• This paper does not report original code.
• Any additional information required to reanalyze the data reported in this paper is available from the lead contact upon request.

### Experimental model and study participant details

Vector cloning, mutagenesis and propagation was performed using DH5α *E. coli* (ThermoFisher), and protein expression was done using BL21 codon plus *E. coli* (Agilent). Female HeLa cells (unauthenticated) were cultured in DMEM with high glucose, L-glutamine and sodium pyruvate (Wisent) supplemented with 10% (v/v) FBS (Sigma) and 1% (v/v) penicillin/streptomycin (Wisent) at 37°C with 5% CO_2_/95% air. No plant, animal or human experiments were performed.

### Method details

#### Purification of the MCUb amino terminal domain (NTD)

The nucleotide sequence encoding the NTD of H. sapiens MCUb (GenBank: NP_060388.2) was cloned into a pET-28a vector using the NheI and XhoI restriction sites. Transformed BL21(DE3) codon plus *E. coli* cells were grown in Luria-Bertani (LB) medium containing kanamycin (60 μg mL^−1^) at 37°C until the optical density (OD) at 600 nm reached ∼0.6–0.8. Subsequently, 0.35 mM of isopropyl β-D-1-thiogalactopyranoside (IPTG) was added to induce 6×His-MCUb_58-159_ expression over 16 h at 24.5°C. Harvested cells were lysed by sonication in 20 mM Tris (pH 8.5), 150 mM NaCl, 1 mM DTT. Purification was performed as described in the nickel-nitrilotriacetic acid (Ni^2+^-NTA) agarose beads manufacturer protocol (HisPur, Thermo Fisher). The 6×His tags were removed by overnight incubation with ∼1 Unit of bovine thrombin (EMD millipore) per mg of protein. Size-exclusion chromatography (SEC) through a Superdex 200 10/300 GL (Cytiva), connected to an AKTA pure FPLC system (Cytiva) at 10°C, was performed as the final purification step in 20 mM Tris (pH 8.5), 150 mM NaCl, 1 mM DTT. The protein concentration of MCUb_58-159_ was estimated using an extinction coefficient (280 nm) of 0.4280 (mg/mL)^−1^ cm^−1^.

#### Purification of the MCU-NTD

The pET-28a MCU_72-189_ vector and associated D147R mutant were from the previously described work.^33^ WT or D147R MCU_72-189_ constructs were transformed in BL21 (DE3) codon plus *E*. *coli*. Transformed cells were grown in LB medium containing kanamycin (60 μg mL^−1^) at 37°C to an OD 600 nm of ∼0.6–0.8, and 200 μM of IPTG was added to induce expression over a 16 h period. The harvested cells were purified under denaturing conditions using (Ni-NTA) resin (HisPur) according to the manufacturer instructions. The protein was refolded in 20 mM Tris, 150 mM NaCl and 1 mM DTT, pH 8.8 by dialysis. After overnight thrombin cleavage (∼1 U/mg protein), the protein was further purified by SEC through a Superdex 200 10/300 GL (Cytiva) connected to an AKTA pure FPLC system (Cytiva) at 10°C. This final purification step buffer was 20 mM Tris (pH 8.5), 150 mM NaCl, 1 mM DTT. The protein concentration of MCU_72-189_ was estimated using an extinction coefficient (280 nm) of 0.3693 (mg/mL)^−1^ cm^−1^.

#### SEC with in-line multi-angle light scattering (MALS)

All protein samples were centrifuged at 9,500×g for 5 min at 4°C immediately before injecting into a 100 μL sample loop. SEC-MALS was performed using a 16 angle Dawn Heleos II light-scattering instrument and Optilab TrEX differential refractometer (Wyatt Technologies) connected in-line with a Superdex 200 Increase 10/300 GL column (Cytiva), controlled by an AKTA Pure FPLC (Cytiva) housed at ∼10°C. Molecular weight was calculated using the ASTRA software (v7.1.4, Wyatt) based on the Zimm plot analysis and using a protein refractive index increment, dn dc^−1^ = 0.185 L g^−1^. Proteins from elution factions were visualized using 15% (w/v) sodium dodecyl sulfate polyacrylamide gel electrophoresis (SDS-PAGE) gels and Coomassie blue staining. All experiments performed in the presence of divalent cations used 5 mM CaCl_2_ or 5 mM MgCl_2_.

#### Circular dichroism (CD) spectroscopy

CD spectra and thermal melts were acquired on a Jasco J-810 Spectropolarimeter equipped with a Peltier temperature control system using a quartz cell with a path length of 0.1 cm. Each spectrum was an average of 3 scans acquired between 200 and 240 nm at a speed of 20 nm min^−1^, data pitch of 1 nm and response time of 8 s. All spectra were corrected for buffer contributions. Thermal melts were acquired by monitoring the change in CD ellipticity at 222 nm while heating from 20°C to 95°C at a rate of 1°C min^−1^, with a data pitch of 1°C and a response time of 8 s. The apparent midpoints of temperature denaturation (T_m_) showing maximum normalized change in CD signal were extracted from the data using two transition Boltzmann sigmoidal fitted curves.

#### 8-Anilinonapthalene 1-sulfonic acid (ANS) binding

Extrinsic ANS (Sigma) fluorescence was assessed using a Cary Eclipse spectrofluorimeter (Varian/Agilent) temperature equilibrated at 22.5°C. The extrinsic ANS fluorescence emission spectra were acquired from 400 nm to 600 nm using an excitation wavelength of 372 nm. Excitation and emission slit widths were set to 10 nm and 20 nm, respectively, and the photomultiplier tube (PMT) detector was set to 600 V.

#### Dynamic light scattering (DLS)

Dynamic light scattering (DLS) measurements were made on a DynaPro Nanostar (Wyatt) under the control of Dynamics 7.8.1.3 software. Protein samples were centrifuged at 12,000×*g* for 10 min before a 5 μL aliquot of the supernatant was loaded into a JC501 microcuvette (Wyatt). Experiments were conducted at 25°C, and data were acquired as 10 consecutive scans per sample with an averaging time of 5 s. The resultant autocorrelation functions were deconvoluted with the regularization algorithm in the Dynamics software to extract the distribution of hydrodynamic radii (R_h_) for each sample.

#### Bis(sulfosuccinimidyl) suberate (BS-3) crosslinking

BS-3 (Thermo Fisher) reactions at 1 mM concentration were carried out for 90 min in 20 mM HEPES (pH 8.4), 150 mM NaCl, 1 mM DTT at ambient temperature. Crosslinking reactions were quenched with 30 mM Tris (pH 8.0) for 15 min at ambient temperature. All samples were separated on a 15% (w/v) SDS-PAGE gel, and protein bands were visualized by Coomassie blue staining.

#### Steady-state fluorescein fluorescence

Steady-state fluorescein fluorescence emission spectra were measured using the Cary Eclipse spectrofluorimeter, temperature equilibrated at 22.5°C. For fluorescein labeling, protein samples were precipitated using chloroform/methanol. Dried samples were resuspended in 20 mM HEPES (pH 8.0) and incubated with 1 mM fluorescein-methane thiosulfonate (MTS) (Toronto Research Chemicals) for 1 h. Labeled protein was separated from free fluorescein-MTS by two 10,000-fold dialyses in 20 mM Tris (pH 8.5), 150 mM NaCl, 1 mM DTT. Molar concentrations of fluorescein-labeled proteins were determined using an 80,000 cm^−1^ M^−1^ extinction coefficient. Samples were excited at 460 nm and fluorescence emission spectra were collected between 490 and 600 nm with excitation and emission slit widths set to 10 nm and 20 nm, respectively, and the PMT set to 600 V. Prior to the addition of unlabeled titrant, samples were manually mixed with a Pasteur pipette until no further change in fluorescence was observed. Curves were fit in GraphPad Prism to a one-site binding model that takes into account protein concentration.

#### Microscale thermophoresis (MST)

MST experiments were performed on a NanoTemper Monolith NT.115 instrument (NanoTemper Technologies). The protein was labeled with the RED-NHS labeling kit (NanoTemper) according to the manufacturer protocol. Samples were loaded into standard glass capillaries (NanoTemper) and the MST data were collected at 22°C using medium MST power and 50–60% excitation power using the Nano-red filters. One site binding analyses were performed using the MO.Affinity Analysis software (v2.3, NanoTemper) and GraphPad Prism.

#### Solution nuclear magnetic resonance (NMR) spectroscopy

Uniformly ^15^N-labeled MCU_72-189_ and ^15^N-labeled MCUb_58-159_ were expressed and purified exactly as described for unlabeled samples with the exception that M9 minimal medium [42 mM Na_2_HPO_4_, 22 mM KH_2_PO_4_, 8.6 mM NaCl, pH 7.4, 0.2% (w/v) D-glucose, 0.2 mg mL^−1 15^N-NH_4_Cl, 1 mM MgSO_4_, 1 μM CaCl_2_, 50 μM thiamine, and 1 μg mL^−1^ biotin] was used during growth and expression. ^1^H-^15^N-heteronuclear single quantum coherence (HSQC) spectra were acquired on a Varian/Inova 600 MHz NMR spectrometer equipped with a triple resonance HCN cryogenic probe using an 8,000 Hz ^1^H sweep width, 1,800 Hz ^15^N sweep width, 24–48 transients, 64 increments in the ^15^N dimension and 1,024 points in the ^1^H dimension. All samples contained 60 μM 4,4-dimethyl-4-silapentane-1-sulfuric acid (DSS), 10% (v/v) D_2_O, and all NMR data were processed using NMRPipe (v10.9).^66^ The total chemical shift perturbations (CSPs) in the ^1^H and ^15^N dimensions for each amide peak was calculated as CSP = $\sqrt{\left[\Delta H^{2}+{\left(0.14\times \Delta N\right)}^{2}\right]}$, where ΔH = ppm change in the ^1^H proton dimension and ΔN = ppm change in the ^15^N nitrogen dimension. Spectra derived from titrations were processed and analyzed using the two-dimensional HSQC spectral series scripts in NMRPipe (v10.9).^66^ Changes in free and bound peak intensities were independently fit to a one-site binding model taking into account protein concentration. The equilibrium dissociation constant (K_D_) was globally shared during simultaneous fits of multiple peak changes in each of free and bound curves.

#### MCU-eGFP and MCUb-mCherry co-localization

HeLa cells were cultured in DMEM with high glucose (Wisent) supplemented with 10% (v/v) FBS (Sigma-Aldrich) and 1% (v/v) penicillin/streptomycin (Wisent) at 37°C with 5% CO_2_/95% air. Cells in 35 mm dishes were transfected with PolyJet (SignaGen) according to manufacturer guidelines. Co-transfection ratios were 0.9 μg:0.1 μg or 0.5 μg:0.5 μg MCUb-mCherry:MCU-eGFP for FRET and Manders analyses, respectively. The pCMV-MCU-eGFP plasmid was from our previous work.^33^ The pcDNA-MCUb-mCherry plasmid was created by cloning mCherry out of pCMV-mCherry-STIM1^65^ into a unique C-terminal EcoRI site introduced into pcDNA-MCUb (Genescript) by site directed mutagenesis. The pcDNA-MCUb-ΔNTD-mCherry construct was generated by introducing an XbaI site at A159 of pcDNA-MCUb-mCherry by site directed mutagenesis. Used in conjunction with the endogenous XbaI site at S77/R78 already in place, the cDNA sequence encoding residues 79–160 was excised by digestion and the plasmid re-ligated.

FRET measurements were made at ∼18–24 h post-transfection. Cells at ∼85–95% confluence (35 mm dish) were washed 2 × 1 mL with Tris-buffered saline (TBS) and trypsinized with 200 μL of 0.25% (w/v) trypsin (Wisent). After ∼5 min, cells were collected, pelleted, washed with 1 × 15 mL of TBS, re-pelleted, suspended in 2 mL of TBS and transferred into a 3 mL stirred cuvette. A PTI QuantMaster spectrofluorometer (Horiba) equipped with electronic temperature control set to 22°C and excitation and emission slit widths set to 12.5 nm and 5 nm, respectively, was used. Emission spectra between 488 and 750 nm (eGFP-donor and mCherry-acceptor spectra) and 570–750 nm (total mCherry spectra) were acquired using excitation wavelengths of 460 and 545 nm, respectively. To minimize light scattering contribution, 495 nm and 570 nm longpass filters (Edmund Optics) were used. Background spectra were collected from cells transfected with empty pCMV vector, and standard curves of 510:545 nm emission ratio intensities were generated from summed individual background and eGFP spectra and used to interpolate eGFP levels from measured spectra containing both eGFP and background signals, where 510 and 545 nm were the peak maxima for known eGFP and background spectra, respectively. The FRET mCherry acceptor emission spectra were determined by subtracting the spectra of cells expressing eGFP alone from cells co-expressing eGFP and mCherry, normalized such that the peak maxima were centered at 609 nm. The total mCherry fluorescence emission was determined by subtracting the spectra of cells expressing empty pCMV from cells co-expressing eGFP and mCherry. The two channel excitation and two channel emission FRET ratios were taken as the mCherry acceptor fluorescence divided by the total mCherry and eGFP emission fluorescence, respectively.^68^

For fluorescence microscopy analyses, cells grown on 18 × 18 mm glass cover slips (No. 1) were incubated with 250 nM of MitoTracker Red (Thermo Fisher Scientific) for 45 min. Subsequently, cells were washed with 1 mL of PBS and fixed with 4% (w/v) paraformaldehyde (Electron Microscopy Sciences) in PBS. After 15 min incubation in the dark, cells were washed 4 × 1 mL PBS and the coverslips were mounted on glass slides using an 85% (v/v) glycerol, 75 mM Tris (pH 8), 0.5% (w/v) propyl gallate (Sigma-Aldrich) medium. Fluorescence within fixed cells was visualized at 600× magnification using an Olympus BX61 upright microscope. eGFP and mCherry were visualized with DAPI/FITC/Texas Red (green excitation) and Rhodamine/Dil/Cy3 (red excitation) filter sets (Chroma), respectively. Using Fiji,^67^^,^^69^ MCU-eGFP and MCUb-mCherry or MCUb-ΔNTD-mCherry RGB images were converted to 16 bit. Background was subtracted using a rolling ball radius of 240 pixels and a binary mask was added. The contrast was enhanced with normalization and 0.35% pixel saturation on a duplicated image. The duplicated image was smoothed and the pixel threshold was determined before generating a new image that included only pixels within the binary mask also found in the original image. Manders’ M1 (red to green pixel co-occurrence) and M2 (green to red pixel co-occurrence) coefficients were then determined using Just Another Co-Localization Plugin (JaCoP) with Costes’ automatic threshold set to equivalent values for both eGFP and mCherry.^70^ Note that the FRET data and the co-localization assessments were performed with different samples. Nevertheless, since the pore forming subunits of the MCU complex directly interact,^11^^,^^44^^,^^45^ both experiments were designed to assess the direct interactions between the two over-expressed pore-forming subunits in the cellular context.

#### GCaMP6f mitochondrial Ca^2+^ uptake measurements

HeLa cells were cultured on 35 mm No. 1S glass bottom dishes (Matsunami) as described for the co-localization experiments. pEGFP-MCU-GCaMP6f, pEGFP-MCUb-GCaMP6f, pEGFP-MCUb-ΔNTD-GCaMP6f constructs were created by cloning MCU, MCUb and MCUb-ΔNTD constructs out of the pcDNA vectors into a pEGFP-N1 using HindIII and KpnI sites. The C-terminal EGFP of these vectors were replaced with GCaMP6f from the pCMV-mito-4x-GCaMP6f vector^64^ using KpnI and MfeI. Transfection of 1 μg of the resultant pEGFP-MCU-GCaMP6f, -MCUb-GCaMP6f or -MCUb-ΔNTD-GCaMP6f was done with PolyJet (SignaGen). Approximately 24 h post-transfection, the dish was visualized on a Nikon Diaphot microscope equipped with a 40× objective (Nikon Fluor 40/1.30 Oil Ph4) to locate a cluster of ∼2–5 GCaMP6f-expressing cells. Fluorescent excitation and emission intensity detection was driven through a Felix Gx software (version 4.9)-controlled PTI RatioMaster RM50 system equipped with a D104 photometer (Horiba). GCaMP6f fluorescence changes were monitored using an excitation wavelength of 470 nm, 0.2 s averaging time and 5 points per s. Excitation slit widths were set to 5 nm. Excitation and emission light passed through a AT/EGFP/FCY2/AF488 filter set (Chroma). Once mounted, the dish was perfused with 3 mL of Ca^2+^-free HEPES buffered saline solution (HBSS) and equilibrated for 10 min. After 60 s of baseline measurement, 3 mL of HBSS supplemented with 2 mM CaCl_2_ and 2.5 μM histamine was perfused through the dish and GCaMP6f fluorescence was measured for an additional 4 min. All traces were exponentially smoothed using a 0.93 dampening factor prior to analysis. At the end of each experiment the cells were perfused with 2 × 10 mL of Ca^2+^-free HBSS to wash out the histamine and incubated for 10 min prior to assessing cells on a different part of the dish. A maximum of three histamine responses per dish were acquired per day and experiments were performed on the same dish for a maximum of two days.

### Quantification and statistical analysis

#### Statistics

Student’s unpaired t-test was applied when comparing two independent groups, paired t-test was applied when comparing the outcome of paired observations on the same samples and a one-way ANOVA followed by Tukey’s post-hoc test was applied when comparing more than two treatment groups. Grubbs single outlier test was used to remove outlying data points. The exact statistical test used, the number of biological replicates and significance value are stated in each respective figure legend. Statistical analyses were performed with GraphPad Prism and R-4.2.1.

## Supplemental information

Document S1. Figures S1‒S3 and Tables S1–S3
