# Supplementary material for: The MCU and MCUb amino-terminal domains tightly interact: mechanisms for low conductance assembly of the mitochondrial calcium uniporter complex
Source: iScience. 2024 Apr 10;27(5):109699. doi: 10.1016/j.isci.2024.109699 (PMC11068563; doi:10.1016/j.isci.2024.109699)
Supplement: Document S1. Figures S1‒S3 and Tables S1–S3 [file mmc1.pdf]

**iScience, Volume *27***

## Supplemental information

# The MCU and MCUb amino-terminal domains tightly interact: mechanisms for low conductance assembly of the mitochondrial calcium uniporter complex

**Megan Noble, Danielle M. Colussi, Murray Junop, and Peter B. Stathopulos**

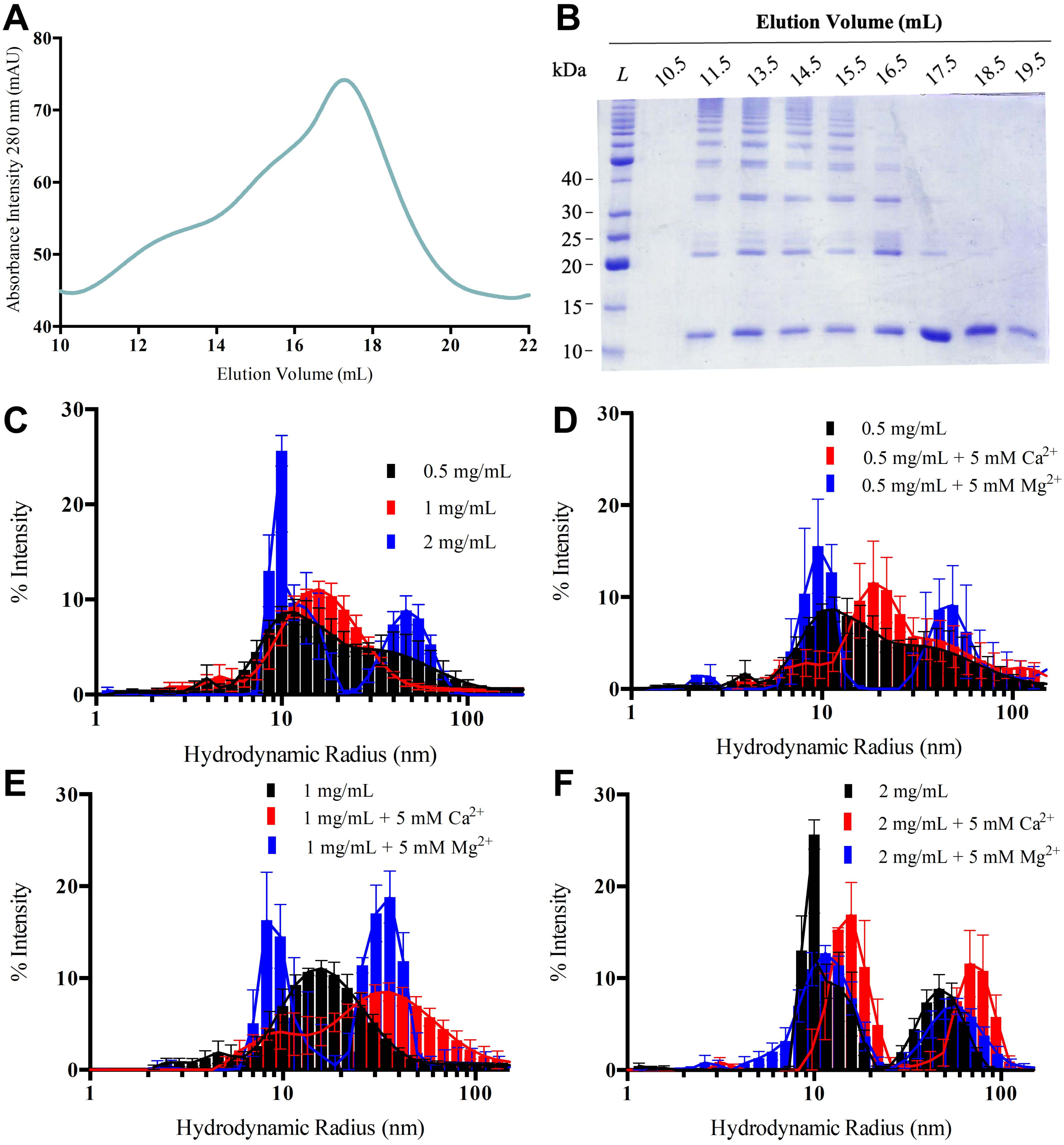

A
Absorbance Intensity 280 nm (mAU)
Elution Volume (mL)
B
Elution Volume (mL)
kDa
L
10.5
11.5
13.5
14.5
15.5
16.5
17.5
18.5
19.5
40
30
25
20
15
10
C
0.5 mg/mL
1 mg/mL
2 mg/mL
% Intensity
Hydrodynamic Radius (nm)
D
0.5 mg/mL
0.5 mg/mL + 5 mM Ca2+
0.5 mg/mL + 5 mM Mg2+
% Intensity
Hydrodynamic Radius (nm)
E
1 mg/mL
1 mg/mL + 5 mM Ca2+
1 mg/mL + 5 mM Mg2+
% Intensity
Hydrodynamic Radius (nm)
F
2 mg/mL
2 mg/mL + 5 mM Ca2+
2 mg/mL + 5 mM Mg2+
% Intensity
Hydrodynamic Radius (nm)


Fig. S1

**Fig. S1** (related to Fig. 4)**. SEC elution profiles and hydrodynamic radii of MCUb-NTD as a function of protein and divalent cation concentration**. **(A)** SEC elution profile through an S200 10/300 GL column monitored by absorbance at 280 nm ($A_{280}$; mAU, milli-absorbance units). **(B)** Coomassie blue staining of MCUb-NTD from elution fractions from *A)*, separated on a 15 % (w/v) SDS-PAGE gel. Higher-order bands represent SDS-PAGE resistant MCUb-NTD oligomers. "*L*" denotes protein ladder. **(C)** Distribution of MCUb-NTD hydrodynamic radii measured at 0.5 mg/mL (black bars), 1 mg/mL (red bars) and 2 mg/mL (blue bars) protein concentration. **(D)** Distribution of MCUb-NTD hydrodynamic radii at 0.5 mg/mL in the absence (black bars) and presence of 5 mM $CaCl_2$ (red bars) and 5 mM $MgCl_2$ (blue bars). **(E)** Distribution of MCUb-NTD hydrodynamic radii at 1 mg/mL in the absence (black bars) and presence of 5 mM $CaCl_2$ (red bars) and 5 mM $MgCl_2$ (blue bars). **(F)** Distribution of MCUb-NTD hydrodynamic radii at 2 mg/mL in the absence (black bars) and presence of 5 mM $CaCl_2$ (red bars) and 5 mM $MgCl_2$ (blue bars). In *C) – F)*, size distributions were deconvoluted from the autocorrelation functions using the regularization algorithm. Solid lines connect the mean total intensity contribution of each hydrodynamic radius. All experiments were carried out in 20 mM Tris (pH 8.5), 150 mM NaCl, 1 mM DTT at 25 °C. Data are means ± SEM of n=3 experiments.

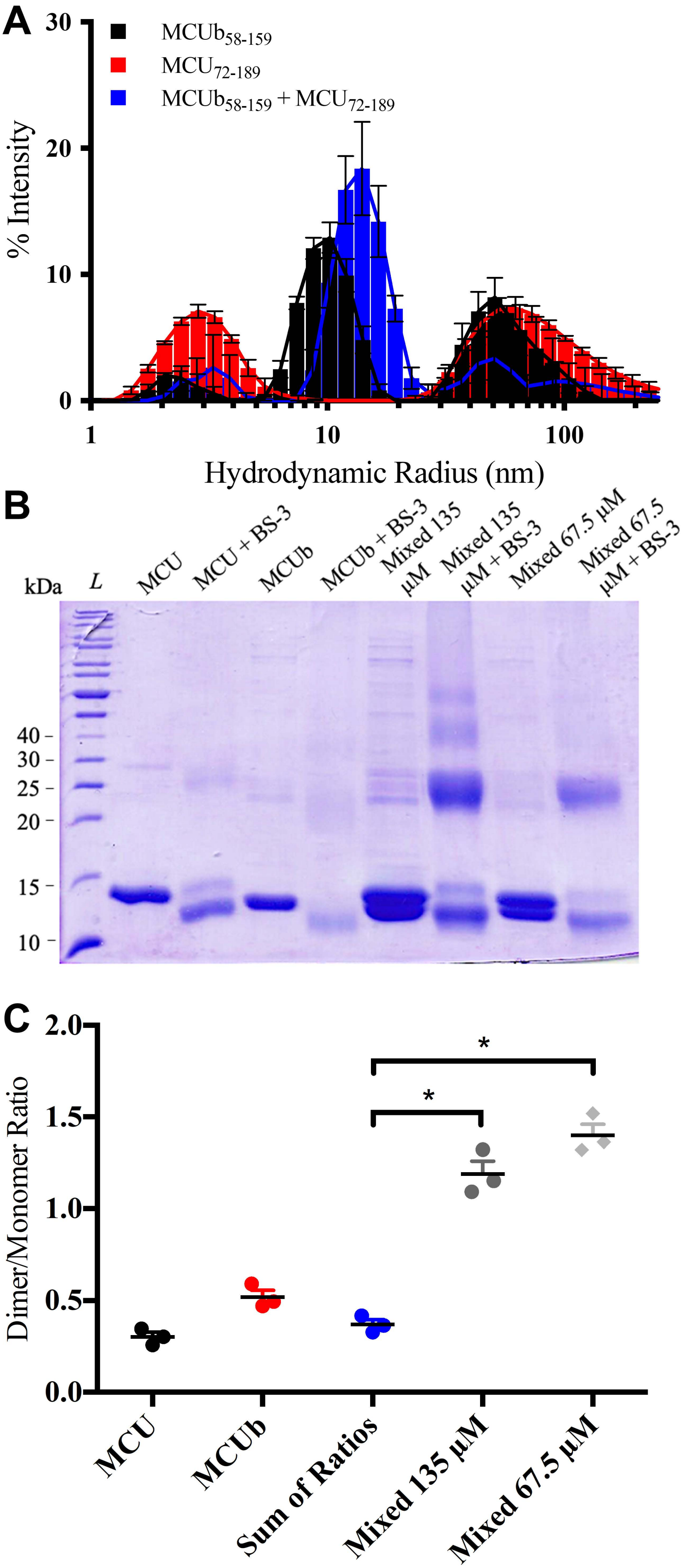

A
% Intensity
MCUb58-159
MCU72-189
MCUb58-159 + MCU72-189
Hydrodynamic Radius (nm)
B
kDa
L
MCU
MCU + BS-3
MCUb
MCUb + BS-3
Mixed 135 μM
Mixed 135 μM + BS-3
Mixed 67.5 μM
Mixed 67.5 μM + BS-3
C
Dimer/Monomer Ratio
MCU
MCUb
Sum of Ratios
Mixed 135 μM
Mixed 67.5 μM


Fig. S2

**Fig. S2 (**related to Fig. 5**). Hydrodynamic radii and BS-3 crosslinking of MCUb-NTD:MCU-NTD complexes. (A)** Distribution of hydrodynamic radii of 67.5 μM MCUb-NTD (black bars), 67.5 μM MCU-NTD (red bars) and mixed MCUb-NTD + MCU-NTD at 67.5 μM each (blue bars) (*i.e.* 135 μM total protein concentration). Solid lines connect the mean total intensity contribution from each hydrodynamic radius. Size distributions were deconvoluted from the autocorrelation functions using the regularization algorithm. **(B)** Coomassie blue-stained SDS-PAGE of MCU-NTD at 67.5 μM, MCUb-NTD at 67.5 μM and mixed MCU-NTD + MCUb-NTD at 67.5 μM each (*i.e.* 135 μM total protein concentration), with and without BS-3 treatment. "*L*" denotes protein ladder. **(C)** Dimer:monomer intensity ratios of BS-3 treated data shown in *B)*. The sum of ratios was calculated from the in-isolation intensities as ($MCU_{dimer}$ + $MCUb_{dimer}$)/($MCU_{monomer}$ + $MCUb_{monomer}$). Intensities were extracted with ImageJ software using the Gel Analysis routine. All experiments were carried out in 20 mM HEPES (pH 8.4), 150 mM NaCl, 1 mM DTT at 22 °C. The image in *B)* is representative and data in *C)* are means ± SEM of n=3 experiments. Data was analyzed using a one-way ANOVA followed by Tukey's post-hoc test (*$p < 0.0001$).

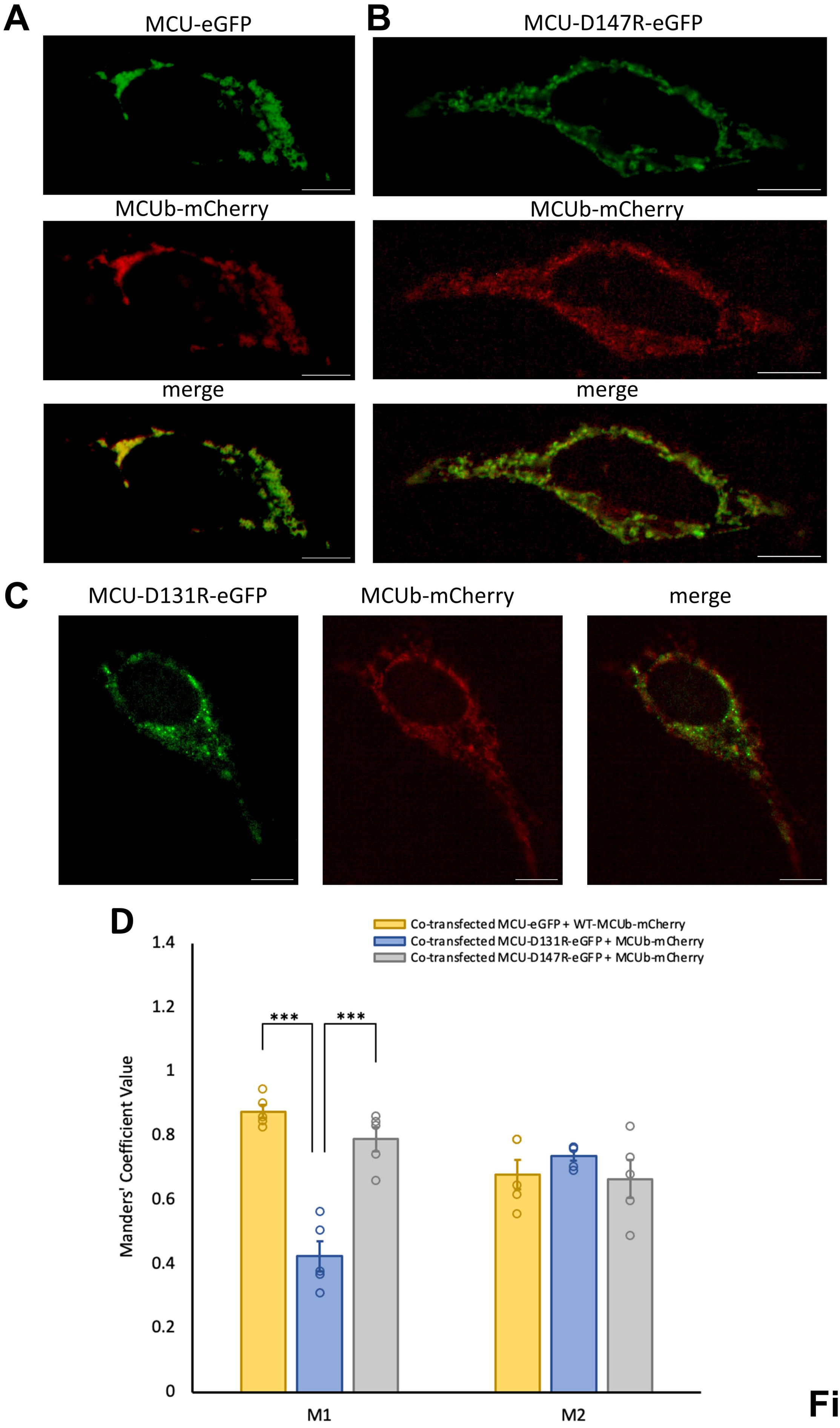
A
MCU-eGFP
MCUb-mCherry
merge
B
MCU-D147R-eGFP
MCUb-mCherry
merge
C
MCU-D131R-eGFP
MCUb-mCherry
merge
D
Co-transfected MCU-eGFP + WT-MCUb-mCherry
Co-transfected MCU-D131R-eGFP + MCUb-mCherry
Co-transfected MCU-D147R-eGFP + MCUb-mCherry
Manders' Coefficient Value
1.4
1.2
1
0.8
0.6
0.4
0.2
0
***
***
M1
M2

Fig. S3

**Fig. S3** (related to Fig. 8)**. MRAP-dependent co-localization of MCU and MCUb in HeLa cells. (A)** Localization of co-transfected MCUb-mCherry (red) and MCU-eGFP (green) in fixed HeLa cells. The merged green and red channels (merge) highlights regions of co-localization (yellow). **(B)** Localization of co-transfected MCUb-mCherry (red) and MCU-D147R-eGFP (green) in fixed HeLa cells. The merged green and red channels (merge) highlights regions of co-localization (yellow). **(C)** Localization of co-transfected MCUb-mCherry (red) and MCU-D131R-eGFP (green) in fixed HeLa cells. The merged green and red channels (merge) highlights regions of co-localization (yellow). **(D)** Manders' M1 and M2 coefficients assessing the co-occurrence of red to green pixels and green to red pixels, respectively, for the co-transfected MCU-eGFP + MCUb-mCherry (yellow), MCU-D131R-eGFP + MCUb-mCherry (blue) and MCU-D147R + MCUb-mCherry (grey) cells. White scale bars in *A) – C)* represent 10 μm. Data in *D)* are expressed as means ± SEM from n=5 cells co-expressing MCUb-mCherry and MCU-eGFP (WT or mutant) from 2-3 separate co-tranfections. Statistical comparison was One way ANOVA followed by Tukey's post-hoc test (***$p < 0.001$).

**Table S1** (related to Fig. 2 and Fig. 5)**.** Secondary structure and thermal stability summary of isolated and mixed MCUb-NTD and MCU-NTD.

| **Analysis** | **Construct** | **Protein Concentration** (mg mL$^{-1}$) | **$CaCl_2$ (5 mM)** | **$MgCl_2$ (5 mM)** | [b]**MRE at 218 nm** (deg cm$^2$ dmol$^{-1}$) (×10$^{-3}$) | [b]**Apparent $T_m$ (°C)** | [c]**$\Delta T_m$ (°C)** |
|---|---|---|---|---|---|---|---|
| Protein concentration | MCUb-NTD | 0.25 | - | - | -6.7 ± 0.1 | 92.0 ± 1.5 | - |
| | MCUb-NTD | 0.5 | - | - | -6.0 ± 0.1 | 86.5 ± 1.4 | -5.5 |
| | MCUb-NTD | 1.0 | - | - | -4.6 ± 0.2 | 75.8 ± 0.5 | -16.2 |
| Divalent cations | MCUb-NTD | 0.5 | - | - | -5.8 ± 0.2 | 89.4 ± 0.3 | - |
| | MCUb-NTD | 0.5 | + | - | -5.9 ± 0.1 | 80.5 ± 0.2 | -8.9 |
| | MCUb-NTD | 0.5 | - | + | -5.7 ± 0.1 | 73.4 ± 0.5 | -16.0 |
| [a]MCUb-NTD + MCU-NTD | MCUb-NTD | 0.5 | - | - | -6.0 ± 0.1 | 78.4 ± 2.8 | - |
| | MCU-NTD | 0.5 | - | - | -4.7 ± 0.2 | 67.4 ± 1.3 | -11.0 |
| | MCUb-NTD + MCU-NTD | 0.5 | - | - | -6.2 ± 0.1 | 82.3 ± 0.9 | 3.9 |

[a]Samples were mixed at 0.5 mg mL$^{-1}$ each such that the final concentration was 0.25 mg mL$^{-1}$ for each component.
[b]Data are means ± SEM of n=3 experiments.
[c]$\Delta T_m = T_{m,\ measured} - T_{m,\ reference}$, where the 0.25 mg mL$^{-1}$, no divalent cation and isolated MCUb-NTD $T_m$ are reference temperatures for the protein concentration, divalent cation and mixed rows, respectively.

**Table S2** (related to Fig. 4)**.** SEC-MALS analysis summary of isolated and mixed MCUb-NTD and MCU-NTD.

| Construct | [Protein] (mg mL$^{-1}$) | $CaCl_2$ (5 mM) | $MgCl_2$ (5 mM) | [a]MALS Determined Weight (kDa) | [b]Stoichiometry | Peak Elution Volume (mL) |
|---|---|---|---|---|---|---|
| MCUb-NTD | 2.0 | - | - | 11.4 ± 0.1 | 0.95 | 17.50 |
| MCUb-NTD | 3.0 | - | - | 11.6 ± 0.3 | 0.97 | 17.55 |
| MCUb-NTD | 4.0 | - | - | 12.2 ± 0.1 | 1.02 | 17.54 |
| MCUb-NTD | 3.0 | + | - | 11.0 ± 0.1 | 0.92 | 17.59 |
| MCUb-NTD | 3.0 | - | + | 11.0 ± 0.1 | 0.92 | 17.57 |
| MCU-NTD | 3.0 | - | - | 13.6 ± 0.2 | 0.98 | 17.14 |
| MCU-NTD | 3.0 | + | - | 13.3 ± 0.1 | 0.96 | 17.16 |
| MCU-NTD | 3.0 | - | + | 14.6 ± 0.1 | 1.05 | 17.12 |
| [c]MCUb-NTD + MCU-NTD | 3.0 | - | - | 21.7 ± 0.4 | 1.68 | 16.36 |
| [c]MCUb-NTD + MCU-NTD | 3.0 | + | - | 22.2 ± 0.1 | 1.72 | 16.40 |
| [c]MCUb-NTD + MCU-NTD | 3.0 | - | + | 20.8 ± 0.1 | 1.61 | 16.35 |

[a]Data are means ± SEM of n=3 experiments.
[b]Stoichiometries of MCUb-NTD, MCU-NTD and MCUb-NTD + MCU-NTD were calculated by dividing the MALS-determined molecular weights by the theoretical monomer molecular weights of 12.0098, 13.8637 and (12.0098+13.8637)/2 kDa, respectively.
[c]Samples were mixed at 3 mg mL$^{-1}$ each such that the final concentration was 1.5 mg mL$^{-1}$ for each component.

**Table S3** (related to Fig. 6 and Fig. 7)**.** Equilibrium dissociation constant ($K_D$) summary for the MCUb-NTD:MCU-NTD interactions.

| Technique | Label | [Label] (μM) | Titrant | Buffer Conditions | Temperature (°C) | $K_D$ (μM) |
|---|---|---|---|---|---|---|
| Steady-state fluorescence | Fluorescein-MCU-NTD | ~0.1 | MCUb-NTD | 20 mM Tris (pH 8.5), 150 mM NaCl, 1 mM DTT, 0.0001% (v/v) NP-40 | 22.5 | [a]0.13 ± 0.02 |
| Steady-state fluorescence | Fluorescein-MCU-NTD | ~0.1 | MCUb-NTD | 20 mM Tris (pH 8.5), 150 mM NaCl, 1 mM DTT, 0.0001% (v/v) NP-40, **5 mM $CaCl_2$** | 22.5 | [a]0.09 ± 0.02 |
| Steady-state fluorescence | Fluorescein-MCU-NTD | ~0.1 | MCUb-NTD | 20 mM Tris (pH 8.5), 150 mM NaCl, 1 mM DTT, 0.0001% (v/v) NP-40, **5 mM $MgCl_2$** | 22.5 | [a]0.16 ± 0.01 |
| Steady-state fluorescence | Fluorescein-MCUb-NTD | ~0.1 | MCU-NTD | 20 mM Tris (pH 8.5), 150 mM NaCl, 1 mM DTT, 0.0001% (v/v) NP-40 | 22.5 | [a]0.13 ± 0.02 |
| Steady-state fluorescence | Fluorescein-MCUb-NTD | ~0.1 | MCU-NTD-D147R | 20 mM Tris (pH 8.5), 150 mM NaCl, 1 mM DTT, 0.0001% (v/v) NP-40 | 22.5 | [a]0.25 ± 0.03 |
| MST | Red-(NHS)-MCUb-NTD | ~0.5 | MCU-NTD | 20 mM Tris (pH 8.5), 150 mM NaCl, 1 mM DTT, 0.05% (v/v) NP-40 | 22 | [a]4.65 ± 1.2 |
| MST | Red-(NHS)-MCUb-NTD | ~0.5 | MCU-NTD-D147R | 20 mM Tris (pH 8.5), 150 mM NaCl, 1 mM DTT, 0.05% (v/v) NP-40 | 22 | [a]11.3 ± 2.0 |
| NMR | $^{15}$N-MCUb-NTD | ~100 | MCU-NTD | 20 mM HEPES (pH 8.4), 150 mM KCl, 1 mM DTT | 20 | [b]0.18 ± 0.13 |
| NMR | $^{15}$N-MCUb-NTD | ~100 | MCU-NTD | 20 mM HEPES (pH 8.4), 150 mM KCl, 1 mM DTT | 20 | [c]0.74 ± 0.97 |
| NMR | $^{15}$N-MCU-NTD | ~100 | MCUb-NTD | 20 mM HEPES (pH 8.4), 150 mM KCl, 1 mM DTT, 1 mM CHAPS | 35 | [b]0.15 ± 0.26 |
| NMR | $^{15}$N-MCU-NTD | ~100 | MCUb-NTD | 20 mM HEPES (pH 8.4), 150 mM KCl, 1 mM DTT, 1 mM CHAPS | 35 | [c]1.25 ± 1.3 |

[a]Data are means ± SEM from n=3 separate experiments.
[b]Data are globally shared values ± SE from simultaneous fits of decreased peak intensity (6-7 peaks) (free state).
[c]Data are globally shared values ± SE from simultaneous fits of increased peak intensity (6-7 peaks) (bound state).
Note: the fluorescein and MST experiments suffered from protein adhesion to materials; the NMR experiments are advantageous since they do not rely on bulky external fluorophores.
